# Intrinsic Conformational Dynamics of Glycine and Alanine in Polarizable Molecular Dynamics Force Fields: Comparison to Spectroscopic Data

**DOI:** 10.1021/acs.jpcb.4c02278

**Published:** 2024-06-15

**Authors:** Brian Andrews, Reinhard Schweitzer-Stenner, Brigita Urbanc

**Affiliations:** † Department of Physics, Drexel University, Philadelphia, Pennsylvania 19104, United States; ‡ Department of Chemistry, Drexel University, Philadelphia, Pennsylvania 19104, United States

## Abstract

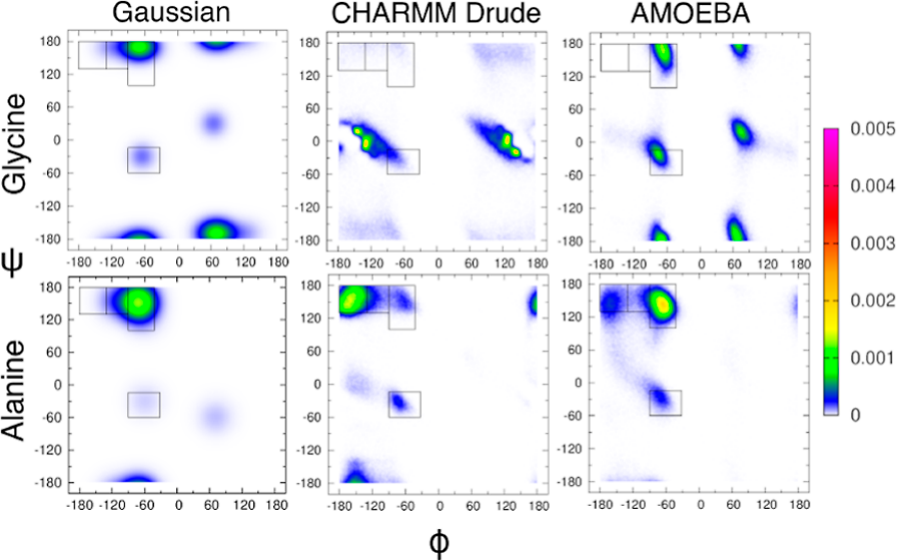

Molecular dynamics (MD) is a great tool for elucidating conformational dynamics of proteins and peptides in water at the atomistic level that often surpasses the level of detail available experimentally. Structure predictions, however, are limited by the accuracy of the underlying MD force field. This limitation is particularly stark in the case of intrinsically disordered peptides and proteins, which are characterized by solvent-accessible and disordered peptide regions and domains. Recent studies show that most additive MD force fields, including CHARMM36m, do not reproduce the intrinsic conformational distributions of guest amino acid residues x in cationic GxG peptides in water in line with experimental data. Positing that a lack of polarizability in additive MD force fields may be the culprit for the reported discrepancies, we here examine the conformational dynamics of guest glycine and alanine residues in cationic GxG peptides in water using two polarizable MD force fields, CHARMM Drude and AMOEBA. Our results indicate that while AMOEBA captures the experimental data better than CHARMM Drude, neither of the two polarizable force fields offers an improvement of the Ramachandran distributions of glycine and alanine residues in cationic GGG and GAG peptides, respectively, over CHARMM36m.

## Introduction

Molecular dynamics (MD) simulations of biomolecules represent a powerful tool in discerning atomistic details of biologically relevant processes such as protein folding, assembly, and interactions of proteins with other organic and inorganic molecules.^1,2^ In MD, interactions among atoms are described by classical empirical potentials, which define the MD force field. The quality of structure predictions relies on the accuracy of the underlying MD force field. As large experimental data sets become available, MD force fields are still developing and improving.^3^ One of the challenges of current MD force fields for proteins is how to capture the dynamics of globular proteins with well-defined native folds arising from funnel-like free energy landscapes^4^ on the one hand and intrinsically disordered proteins (IDPs)^5−7^ with one or more unfolded and solvated peptide regions that populate a large ensemble of equilibrium conformations arising from rugged free energy landscapes^8,9^ on the other hand. In addition to being involved in many important biological processes in a living cell, many IDPs are associated with human diseases, including cancer, cardiovascular disease, amyloidoses, neurodegenerative diseases, and diabetes.^10−15^ Thus, the development of a sufficiently accurate MD force field to allow for reliable atomistic insights into the dynamics of IDPs is of utmost importance.

It is well-documented that additive MD force fields produce overly collapsed IDP conformations.^16,17^ Strengthening the protein–water interactions while keeping the water–water and protein–protein interactions intact^17,18^ and improving the water model both increase protein solubility.^16,19,20^ However, increased protein solubility alone may not be sufficient to resolve a related issue, i.e., an overly favorable protein self-assembly.^21−23^ Another problem arises from the necessity to reproduce the conformational sampling of solvent accessible segments of IDPs. Conformational ensembles of such regions are expected to be governed by context-dependent conformational preferences of amino acid residues in water. These context-dependent conformational preferences depend on the intrinsic propensities, which are modified by nearest-neighbor and possibly even second nearest-neighbor interactions.^24,25^ A good MD force field is expected to capture both the intrinsic and context-dependent conformational preferences of amino acid residues in water.

Extensive spectroscopic data with at least 5 NMR scalar coupling constants as well as amide I′ band profiles from infrared (IR), Raman, and vibrational circular dichroism (VCD) spectra have been utilized to constrain the Ramachandran distributions of 14 guest amino acid residues x in cationic GxG peptides in water, which reflect the intrinsic conformational states of guest residues.^26−32^ To this end, Schweitzer-Stenner developed a Gaussian decomposition model for the Ramachandran distribution of each guest residue x, which is described as a superposition of two-dimensional Gaussian subdistributions associated with ubiquitous conformational states, such as polyproline II (pPII), β-strand, right- and left-handed helices, and various types of turns.^33^ The locations, widths, and relative weights of these subdistributions were then optimized to best fit the residue-specific spectroscopic data described above, resulting in residue-specific Gaussian Ramachandran distributions. Intrinsic conformational propensities for 14 cationic GxG peptides in water were recently used in an assessment study of several additive MD force fields (CHARMM36m,^19^ Amber ff14SB,^34^ Amber ff19SB,^35^ and OPLS-AA/M^36^) only to reveal a severe lack of residue specificity and/or a rather poor agreement with spectroscopic data as quantified by large reduced χ_*J*_^2^ and χ_VCD_^2^ values, which surpass the corresponding reduced χ_*J*_^2^ and χ_VCD_^2^ values of the benchmark experiment-derived Gaussian Ramachandran distributions by an order of magnitude.^37^

It is particularly surprising that the additive MD force fields fail to reproduce well the intrinsic conformational ensembles of alanine and glycine, the amino acid residues that are frequently used in quantum mechanical (QM) calculations underlying the parameterization of dihedral torsional potentials in MD force fields. Evaluating six MD force fields, including Amber ff14SB, CHARMM36m, and OPLS-AA/M, with respect to their ability to reproduce the available spectroscopic data for the central alanine residue in cationic GAG and AAA peptides, Zhang et al. showed that Amber ff14SB combined with the TIP3P water model performed better than the other force fields yet resulted in quite high reduced χ_*J*_^2^ and χ_VCD_^2^ values when compared to the corresponding experiment-based Gaussian Ramachandran distributions.^38^ In a follow-up study by Andrews et al., experiment-based Gaussian modeling was applied to the central glycine residue in cationic GGG as a model of a peptide backbone to demonstrate that the pPII state is favored by glycine despite its achiral character.^39^ Andrews and collaborators revealed that the high pPII preference is driven by the tendency of water to maximize the number of hydrogen bonds between water and the functional groups of the central glycine, which supports the hypothesis that the high pPII content shared by several amino acid residues in water stems from the water-backbone hydrogen bonding.^39^ Interestingly, in this case, CHARMM36m and OPLS-AA/M performed better than Amber ff14SB in capturing the intrinsic conformational dynamics of glycine residue in water, yet all additive force fields in these studies produced significantly higher reduced χ_*J*_^2^ and χ_VCD_^2^ values than the corresponding experiment-based Gaussian Ramachandran distributions.^37,39^

We here ask whether the poor agreement of MD-derived Ramachandran distributions of guest residues x in cationic GxG peptides in water with the spectroscopic data described above is due to the limitations of additive MD force fields. Additive MD force fields treat atoms as rigid point-charge particles with electronic degrees of freedom averaged out to increase the computational efficiency and allow for simulations of larger systems on longer time scales. In contrast, polarizable MD force fields have the electronic polarization explicitly embedded into the electrostatic potential. We selected two recently reported polarizable MD force fields: CHARMM Drude^40,41^ with the SWM-NDP4 water model^42,43^ (Drude-2019) and AMOEBA (atomic multipole optimized energetics for biomolecular simulation)^44^ with anisotropically polarized water model^45^ (AMOEBA 2018). While both force fields, CHARMM Drude-2019 and AMOEBA, feature induced dipole calculations, the implementation of this feature is very different in the two force fields. The induced electronic polarizability is implemented in Drude-2019 through explicit Drude particles modeled as the classical Drude oscillators, whereas the electrostatic parameters in AMOEBA are derived from residue-specific atomic multipoles using high-level gas-phase QM calculations. In this work, we focus on intrinsic conformational ensembles of guest alanine and glycine in cationic GxG peptides in water. These two residues are elementary as they are used in parameterization of the backbone torsional potentials in MD force fields, including Drude-2019 and AMOEBA; hence, capturing their respective Ramachandran distributions in agreement with spectroscopic data is of paramount importance.

## Methods

### MD Simulations of Tripeptides in Water

Tripeptides GGG and GAG were constructed using the visual molecular dynamics software package.^46^ A single tripeptide was immersed into a 125 nm^3^ cubic box with periodic boundary conditions at a temperature of 300 K using OpenMM 8.0.^47^ Simulations were performed in the CHARMM Drude 2019 force field^40,41^ with the SWM-NDP4 water model^42^ and the AMOEBA force field with its version of the TIP3P water model.^44,45^ In each simulation, the tripeptide under consideration was protonated at the N terminus (NH_3_^+^) and neutral at the C terminus to mimic the acidic pH used in experiments. The carboxyl group (COOH) was used as the neutral C-terminal group in CHARMM Drude, and the neutral CONME capping (with the corresponding CONHCH_3_ group) was used in AMOEBA. A single Cl^–^ ion was added to obtain an electrostatically neutral system. The nonbonded cutoff radius, for both electrostatic and short-range attractive and repulsive interactions, was set to 1 nm. Steepest descent was utilized during the energy minimization for 100,000 time steps. A 1 fs time step was used during the equilibration and production steps. The Drude Langevin integrator^48^ was utilized within CHARMM Drude, whereby the temperature and temperature coupling coefficient were set to 300 K and 1 ps^–1^ for regular atoms, respectively, and 1 K and 10 ps^–1^ for Drude particles, respectively. Both Thole damping and the hard wall constraint are used in CHARMM Drude simulations.^40^ The maximum distance between an atom’s nucleus and the associated Drude particle was set to 0.02 nm to prevent the polarization catastrophe.^40,49^ The Langevin integrator^50^ was utilized for simulations with AMOEBA with the temperature and temperature coupling coefficient set to 300 K and 1 ps^–1^, respectively. In AMOEBA, mutual polarization with a tolerance of 0.00001 was used to calculate induced dipoles and multipole forces. The Monte Carlo barostat^51^ was used to maintain an average pressure of 1 bar at 300 K with both force fields. Additional simulations of GGG and GAG were performed with NH_3_^+^ and COO^–^ at the N and C termini, respectively, to determine the effects of end groups on the analysis and compare the two force fields using the same C-terminal capping. The preparation of these simulations followed the same protocol as described above aside from the addition of ions because both peptides are electrostatically neutral when using these termini cappings. All production runs were 500 ns long. The equilibration period of production runs is set to 50 ns, and the analysis is done on data extracted from 50 to 500 ns of each MD trajectory.

### Analysis

#### MD-Derived Ramachandran Distributions

Backbone dihedral angle pairs of the central residue of each tripeptide are calculated with the MDTraj package^52^ using time frames within 50–500 ns, sampled every 2 ps, of each MD trajectory. Ramachandran distributions are constructed by subdividing the Ramachandran space into 32,400 bins (2° × 2°) and calculating the respective local per-bin probabilities.

#### Definition of Mesostates

Mesostates are used to compare conformational distributions derived from experimental data and MD simulations. The following mesostate definitions^37^ are used: (a) pPII (−90° < ϕ < −42°, 100° < ψ < 180°), (b) antiparallel β-strand (aβ) (−180° < ϕ < −130°, 130° < ψ < 180°), (c) the transition region between aβ and pPII (βt) (−130° < ϕ < −90°, 130° < ψ < 180°), and (d) right-handed α-helix (−90° < ϕ < −32°, −60° < ψ < −14°). The mesostate populations are calculated from the MD trajectories using time frames 50–300 ns as the number of conformations within each mesostate region normalized by the total number of conformations. Because triglycine is achiral, the (−ϕ, −ψ) conformations are identical to the corresponding (ϕ, ψ) conformations. Consequently, for the central glycine in triglycine, the mesostate populations are obtained by adding the respective left-handed and right-handed populations.

#### J-Coupling Constants and Amide I′ Profiles

The experimental data that we use to assess the MD-derived Ramachandran distributions of guest residues x in GxG peptides in water were reported earlier.^26,28−31^ The experimental data set for each guest residue x encompasses the ϕ-dependent scalar coupling constants ^3^*J*(*H*^N^,*H*^C_α_^), ^3^*J*(*H*^*N*^,*C*^′^),^3^*J*(*C*,*C*^′^)(for glycine),^3^*J*(*H*^*N*^,*C*_β_)(for alanine),^3^*J*(*H*^*C*_α_^,*C*^′^), the ψ-dependent constant ^1^*J*(*N*, *C*_α_), and the amide I′ profiles in the respective IR, Raman, and VCD spectra (the prime sign indicates that the vibrational spectra are acquired for GxG peptides in heavy water, D_2_O).

#### Gaussian Modeling and Gaussian Ramachandran Distributions

The Gaussian model, previously developed by Schweitzer-Stenner,^33^ is used as a benchmark model of the Ramachandran distribution for each guest residue x in GxG peptides. In brief, Gaussian modeling is based on a linear Gaussian decomposition, whereby a Ramachandran distribution is modeled as a superposition of two-dimensional Gaussians associated with distinct local secondary structure states (such as pPII, right- and left-handed α-helix, turns, parallel and antiparallel β, and various turns). The Gaussian model parameters (relative statistical weights of Gaussian subdistributions, their location in the Ramachandran space, and their corresponding widths) are then adjusted to best fit the available *J*-coupling constants and amide I′ band profiles.^33^ The optimization of the Gaussian model parameters is carried out by using the empirical Karplus equations^53−56^ and an exitonic coupling formalism to calculate conformational averages of *J*-coupling constants and amide I′ band profiles, respectively.^24,28−30,33^ In the current study, the Gaussian model Ramachandrans were generated for central glycine and alanine in previous studies.^38,39^ In the evaluation of the MD-derived Ramachandran distributions, the same algorithm described above is used to calculate force field-specific MD-derived *J*-coupling constants and amide I′ band profiles. Consistent with MD-derived Ramachandran distributions, Gaussian Ramachandran distributions are also constructed by subdividing the Ramachandran space into 32,400 bins (2° × 2°) and calculating the respective local per-bin probabilities.

#### Reduced χ^2^ Functions

We use a reduced χ_*J*_^2^ function to quantitatively assess the ability of MD force fields and the Gaussian model to capture the conformational ensemble of guest residue x in GxG peptides in water
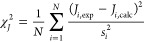
1where *N* is the number of *J*-coupling constants (in our case five), *J*_*i*,exp_ are the experimental *J*-coupling constants, *J*_*i*,calc_ are the calculated *J*-coupling constants obtained from MD-derived or Gaussian Ramachandran distributions, and *s*_*i*_ are the uncertainties due to the reported experimental errors^27^ and the errors associated with the Karplus parameters,^54^ which are combined using the Gaussian error propagation method. Generally acceptable fits are associated with reduced χ_*J*_^2^ values below 2. However, this criterion is applicable only if all errors associated with experimental data are known. Unfortunately, this cannot be guaranteed in the current work because the errors of the Karplus parameters are not available for ^1^*J*(NC_α_) and ^3^*J*(CC′). In such cases, we use the experimental uncertainties alone, which are typically smaller than the propagation errors due to fitting that determines the Karplus parameters, leading to an overestimation of χ_*J*_^2^ values. This shortcoming, however, does not directly affect a comparison of χ_*J*_^2^ values obtained with Gaussian modeling and MD simulations.

In analogy to the reduced χ_*J*_^2^ function, a reduced χ_VCD_^2^ function is defined to evaluate the MD force fields and the Gaussian model with respect to their capacity to reproduce the experimental amide I′ band profile of guest residue x
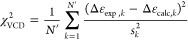
2where *N*′ is the number of wavenumbers from 1600 to 1720 cm^–1^, Δε_exp,*k*_ is the experimental value for all *k* considered, and *s*_*k*_ are standard deviations derived from an analysis of a spectral region dominated by noise. Note that due to the achiral nature of triglycine, the VCD signal for the guest glycine in GGG vanishes. Following our previous work, we calculated χ_VCD_^2^ for the glycine residue by setting Δε_exp,*k*_ = 0 and *s*_*k*_ = 1 for all *k* values.^39^

#### Hydrogen Bonding Analysis

Hydrogen bonds were identified using the MDAnalysis python package.^57,58^ The donor–acceptor distance cutoff was 3 Å, and the donor-hydrogen-acceptor angle was allowed to deviate from a planar bond by 20°. The average number of intrapeptide and peptide–water hydrogen bonds was calculated by counting the number of intrapeptide and water–peptide hydrogen bonds every 2 ps using times 50–500 ns and dividing each total by the number of frames (225,000 frames). Error bars were calculated as the standard error of the mean (SEM) values using 1 ns averages.

## Results

Over the past decade, Schweitzer-Stenner and collaborators have acquired an extensive set of spectroscopic data for guest residues x in cationic GxG peptides in water and developed a Gaussian model of residue-specific Ramachandran distribution that provides the best fit to the spectroscopic data.^26−32^ Recently, several additive MD force fields were evaluated with respect to their capacity to reproduce these spectroscopic data for 14 guest residues x in cationic GxG peptides in water.^37−39^ In these assessment studies, MD-derived Ramachandran distributions were used to calculate the *J*-coupling constants and VCD amide I′ profiles, which were then directly compared to the respective experimental data, resulting in reduced χ_*J*_^2^ and χ_VCD_^2^ values for each residue as a quantitative measure of a deviation from the experimental data. These MD-derived χ^2^ values were compared to the respective values produced by the Gaussian model-derived Ramachandran distributions, revealing that the Gaussian model outperforms the additive MD force fields by at least an order of magnitude (see Table S3 in Andrews et al.^37^). Guest glycine and guest alanine residues in GGG and GAG, respectively, were no exception, although most MD force fields are in part based on QM calculations on alanine dipeptides and other short alanine-based peptides. Considering that the lack of polarizability in the additive MD force fields might be the reason behind the poor agreement with experimental data, we here perform 500 ns-long MD simulations of cationic GGG and GAG in water using two polarizable MD force fields, CHARMM Drude and AMOEBA. The two respective Ramachandran distributions are then used to calculate, for each guest residue, five *J*-coupling constants and the VCD amide I′ profile, such that a direct comparison to spectroscopic data can be made in terms of reduced χ_*J*_^2^ and χ_VCD_^2^ values (see Methods for details). The experiment-based Gaussian model Ramachandran distributions are used as a gold-standard comparison of the reduced χ_*J*_^2^ and χ_VCD_^2^ values.^33^ In addition, we compare the results derived with CHARMM Drude and AMOEBA with the previously reported results obtained within CHARMM36m.^19^

### Intrinsic Conformational Ensemble of the Glycine Residue in Water

**Figure 1:**
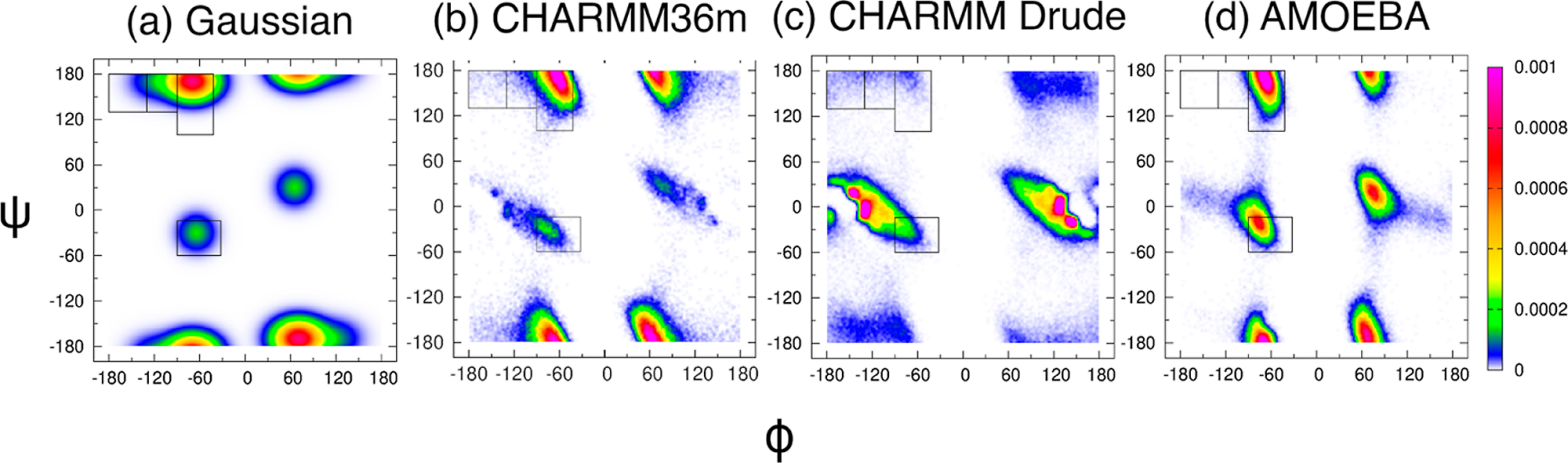
Ramachandran distributions of the central glycine in GGG with neutral C-terminal capping obtained from the (a) Gaussian model, (b) additive force field CHARMM36m, and two polarizable force fields: (c) CHARMM Drude and (d) AMOEBA. The rectangular boxes correspond to the four mesostates as defined in Methods. Distributions (a,b) are reproduced from Andrews and collaborators.^39^ Available under a CC-BY 4.0 license. Copyright 2020 Brian Andrews, Shuting Zhang, Reinhard Schweitzer-Stenner, and Brigita Urbanc.

Figure 1a shows Ramachandran distributions for the central glycine in cationic GGG in water obtained from the experiment-based Gaussian model,^33^ which demonstrates that the central glycine favors the pPII state, as reported previously.^39^ Previous work also showed that CHARMM36m captures the preference of the central glycine for the pPII state relatively well, although not better than the Gaussian model.^39^ The new results in Figure 1c,d are derived from the two polarizable force fields, CHARMM Drude and AMOEBA, respectively, as described in Methods. Mesostate populations, as defined in Methods, are tabulated in Table S1 for reference and comparison. The dihedral angles as a function of simulation time in Figure S1 demonstrate that the conformations are not trapped in any local minima but rather sample the entire allowed Ramachandran space.

**Figure 2:**
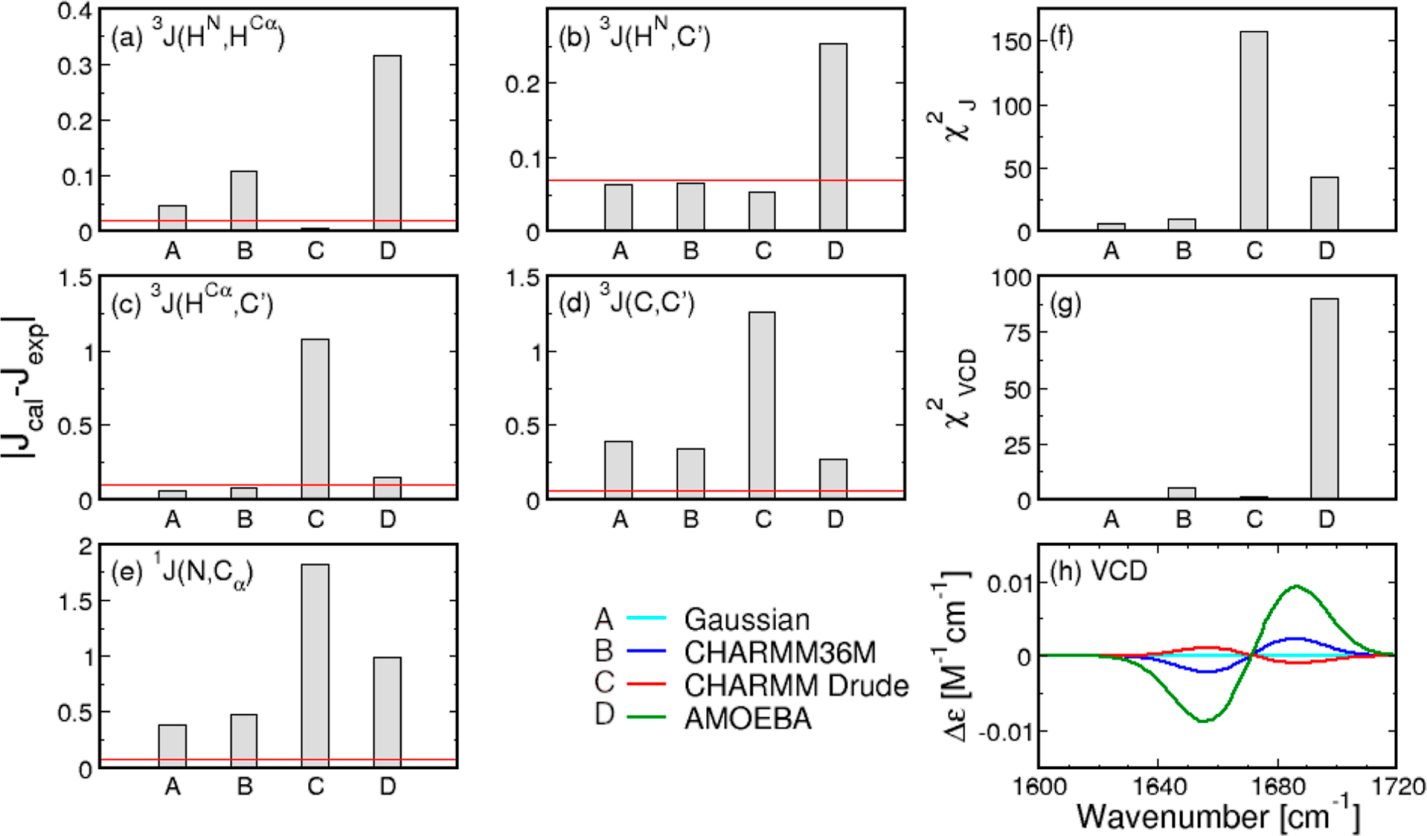
Comparison between experimental and calculated *J*-coupling constants and amide I′ profiles for central glycine in GGG with a neutral C-terminal capping. (a–e) Absolute differences between calculated and experimental values of the five *J*-coupling constants for the Gaussian model and the three MD force fields. Red lines correspond to experimental uncertainties. (f,g) Reduced χ_*J*_^2^ and χ_VCD_^2^ values for the Gaussian model and MD force fields. χ_VCD_^2^ values are multiplied by 10^7^. (h) VCD amide I′ profiles as predicted by the Gaussian model and derived from MD force fields in comparison to experimental data.

The Ramachandran distribution produced by CHARMM Drude shows almost no population in the pPII region (Figure 1c). Instead, the central glycine in GGG populates the region of the Ramachandran space near ψ = 0, i.e., a slightly forbidden region to the left of *i* + 2 type I, II′ β turns. This result is at odds with the spectroscopic data, which are outlined in Table S2. Figure 2 displays the absolute differences between the experimental and calculated (Gaussian model and MD-derived) values for the 5 *J*-coupling constants (Figure 2a–e) as well as experimental versus calculated VCD amide I′ profiles (Figure 2h), alongside the corresponding reduced χ_*J*_^2^ and χ_VCD_^2^ values (Figure 2f,g), demonstrating a very large χ_*J*_^2^ value for CHARMM Drude relative to the Gaussian model and the other two force fields. The source of this large value is a poor reproduction of ^3^*J*(*H*^*C*_α_^,*C*^′^), ^3^*J*(*C*, *C*′), and ^1^*J*(*N*, *C*_α_) values. On the other hand, the inversion symmetry of the Ramachandran distribution for achiral glycine is well accounted for, as demonstrated by a near-vanishing VCD amide I′ profile (Figure 2h) and the corresponding low χ_VCD_^2^ value (Figure 2g).

The AMOEBA-derived Ramachandran distribution for the central glycine in GGG is dominated by the pPII state with a mesostate population of 41% (Table S1), which is qualitatively consistent with the Gaussian model and most additive MD force fields evaluated in previous studies.^37,39^ Relative to the Gaussian model predictions, AMOEBA underestimates the βt mesostate population and overestimates the α-helical mesostate population (Table S1). A direct comparison to the spectroscopic data in Figure 2 shows that the differences between AMOEBA-derived and experimental *J*-coupling constants are larger than the respective differences derived from the Gaussian model and the other force fields for ^3^*J*(*H*^*N*^,*H*^*C*_α_^) and ^3^*J*(*H*^*N*^, *C*′). Additionally, the associated ^1^*J*(*N*, *C*_α_) value deviates from the respective experimental value more than those obtained within the Gaussian model and CHARMM36m simulations. AMOEBA outperforms CHARMM Drude in reproducing the experimental data for glycine, resulting in a lower χ_*J*_^2^ value, but does not perform better than the Gaussian model and CHARMM36m. The AMOEBA-derived VCD amide I′ profile exhibits pronounced peaks, which are above the experimental noise, and the correspondingly large χ_VCD_^2^ value, as shown in Figure 2h,g, respectively.

Figure S2 shows the experimental and calculated isotropic and anisotropic Raman and IR spectra for the central glycine in cationic GGG. The CHARMM Drude-derived Ramachandran distribution produces anisotropic Raman and IR spectra that deviate the most from the experimental data, whereas AMOEBA-derived spectra show smaller deviations from the experimental data that are similar to those obtained from the CHARMM36m-derived Ramachandran distribution. The above findings indicate that neither of the two polarizable force fields reproduces the spectroscopic data for central glycine in cationic GGG in water better than CHARMM36m, and none of the three MD force fields outperforms the Gaussian model.

### Intrinsic Conformational Ensemble of the Alanine Residue in Water

**Figure 3:**
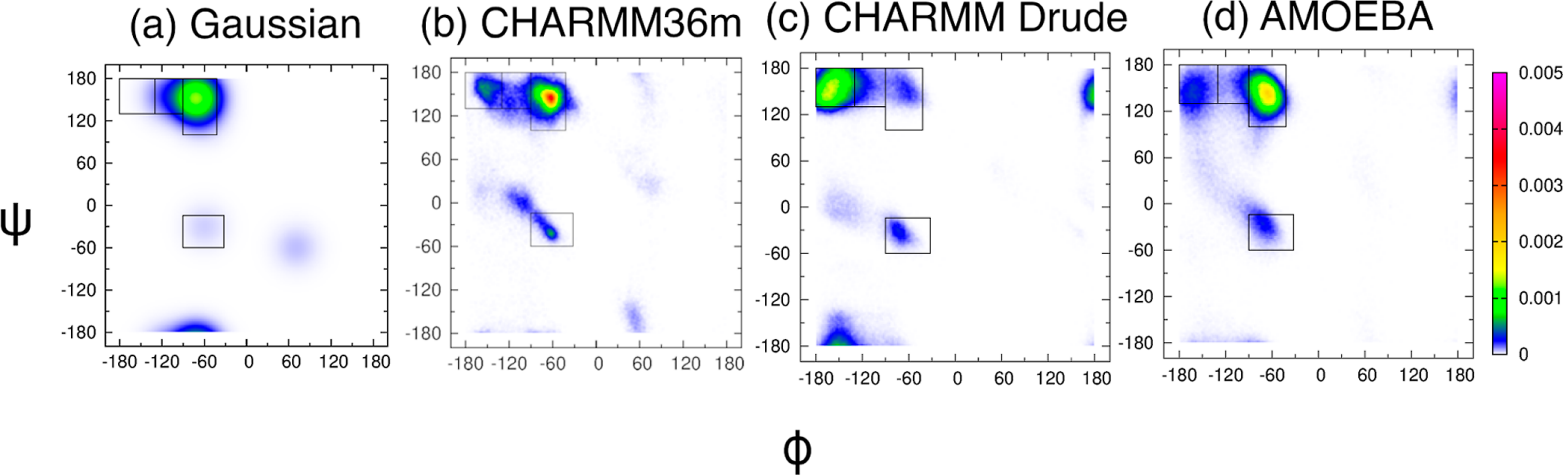
Ramachandran distributions of alanine in cationic GAG with neutral C-terminal capping obtained from the (a) Gaussian model, (b) additive force field CHARMM36m, and two polarizable force fields: (c) CHARMM Drude and (d) AMOEBA. The rectangular boxes correspond to the four mesostates as defined in Methods. Distributions (a,b) are reproduced from Zhang and collaborators^38^. Copyright 2019 American Chemical Society.

Figure 3a shows Ramachandran distributions for the central glycine in cationic GGG in water obtained from the experiment-based Gaussian model,^33^ as reported previously.^38^ Previous work also showed that the Ramachandran distribution derived from CHARMM36m (Figure 3b) qualitatively captures the preference of alanine for the pPII state, although Amber ff19SB outperforms CHARMM36m and OPLS-AA/M, and none of the additive MD force fields performs better than the Gaussian model.^37,38^ The new Ramachandran distributions for alanine in cationic GAG derived from CHARMM Drude and AMOEBA are shown in Figure 1c,d, respectively, and the corresponding mesostate populations are tabulated in Table S1. The dihedral angles as a function of simulation time in Figure S3 show that the entire region of the allowed Ramachandran space is sampled during simulations. The Ramachandran distribution obtained within CHARMM Drude (Figure 3c) is consistent with results reported by Lopes and collaborators.^40^ The CHARMM Drude-derived Ramachandran distribution, unlike those produced by the additive MD force fields,^37,38^ is dominated by aβ conformations with the population of this mesostate of ≈52% (Table S1). The pPII state with the corresponding mesostate population of ≈7% is strongly underestimated in CHARMM Drude relative to the Gaussian model and the other MD force fields (Table S1). The Ramachandran distribution of alanine in cationic GAG produced by AMOEBA is qualitatively comparable to the CHARMM36m-derived Ramachandran distribution. The predominant conformations produced by AMOEBA correspond to the pPII state with a mesostate population of ≈55% (Table S1). The pPII population in AMOEBA is thus slightly underestimated when compared to the pPII population predicted by the Gaussian model. AMOEBA produces an increased population of conformations in the aβ region of the Ramachandran space, which leads to an overestimation of the aβ mesostate population relative to the Gaussian model prediction (Table S1). An overestimation of this mesostate for alanine in cationic GAG in water is common among the additive MD force fields.^37,38^

**Figure 4:**
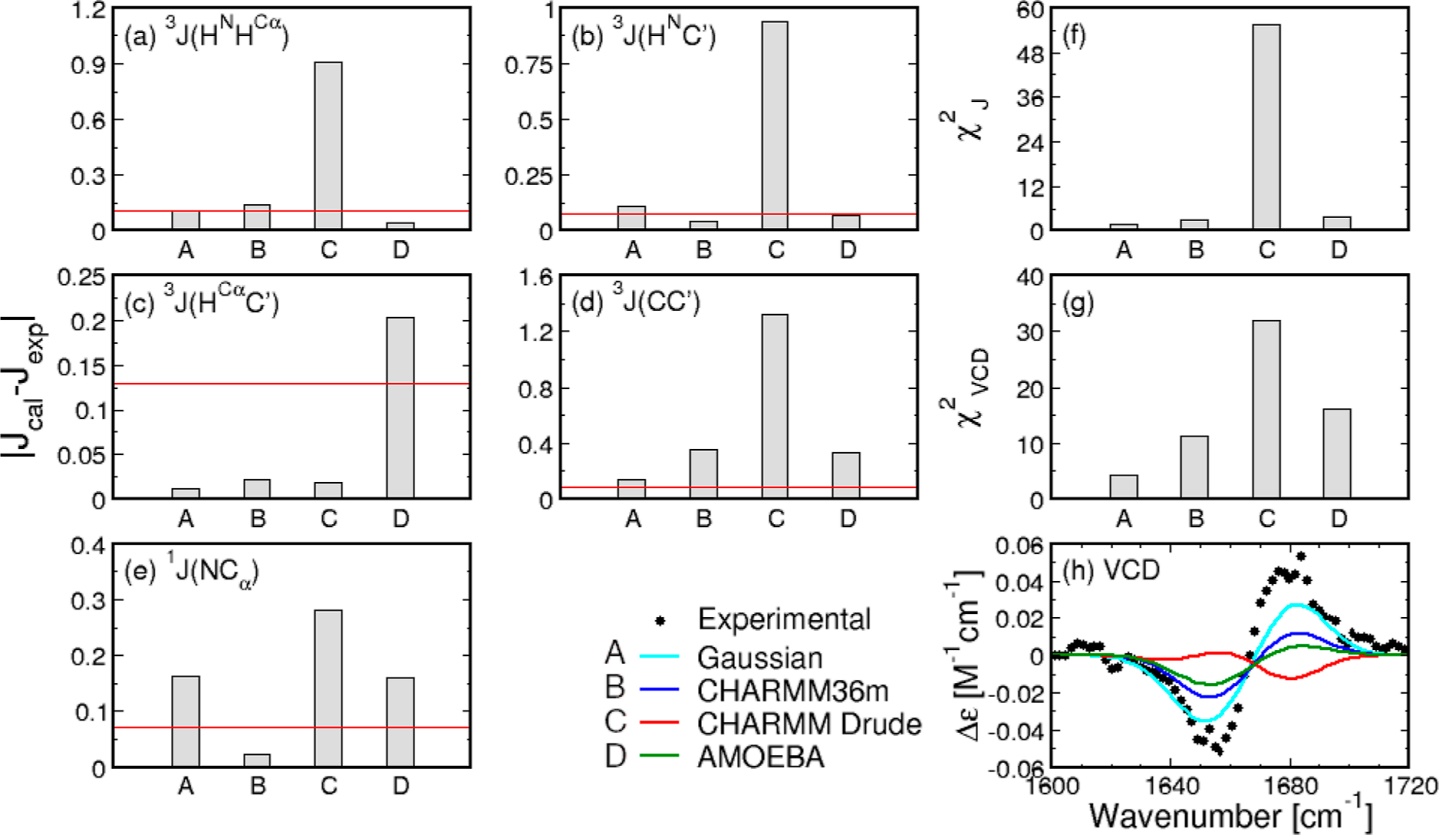
Comparison between experimental and calculated *J*-coupling constants and amide I′ profiles for alanine in cationic GAG with neutral C-terminal capping. (a–e) Absolute differences between calculated and experimental values of the five *J*-coupling constants for the Gaussian model and the three MD force fields. Red lines correspond to the experimental uncertainties. (f,g) Reduced χ_*J*_^2^ and χ_VCD_^2^ values for the Gaussian model and MD force fields. (h) VCD amide I′ profiles as predicted by the Gaussian model and derived from MD force fields in comparison to experimental data.

The values of the experimental, Gaussian model, and MD-derived *J*-coupling constants for alanine of cationic GAG are displayed in Table S2. Figure 4a–e shows the difference between experimental values and calculated values from the Gaussian or MD Ramachandran distributions, and Figure 4f shows the corresponding reduced χ_*J*_^2^ values. The CHARMM Drude Ramachandran distribution of alanine produces the largest χ_*J*_^2^ value, which originates in the largest deviations from the experimental values for all *J*-coupling constants except for ^3^*J*(*H*^*C*_α_^,*C*^′^). AMOEBA-derived *J*-coupling constants deviate from the experimental data comparably to the Gaussian model and CHARMM36m-derived *J*-coupling constants, except for the AMOEBA-derived^3^*J*(*H*^*C*_α_^,*C*^′^) value, which deviates the most from the experimental value. The reduced χ_*J*_^2^ value for AMOEBA is comparable to that produced by CHARMM36m and slightly worse than the one predicted by the Gaussian model.

Figure 4h,g shows the experimental and calculated VCD amide I′ profiles and the associated reduced χ_VCD_^2^ values, respectively. The MD-derived VCD amide I′ profile within the CHARMM Drude force field produces low peak magnitudes with reverse bias when compared to the experimental data and VCD amide I′ profiles calculated from the Gaussian model and derived from the other two force fields. CHARMM Drude produces the largest reduced χ_VCD_^2^ value. The VCD amide I′ profile calculated from the AMOEBA Ramachandran distribution has lower peak magnitudes than that predicted by CHARMM36m and the Gaussian model. Therefore, AMOEBA produces a higher reduced χ_VCD_^2^ value than that of the Gaussian model and CHARMM36m but lower than that of CHARMM Drude. Figure S4 shows the experimental and calculated Raman and IR amide I′ profiles for alanine in cationic GAG in water. The Gaussian model and MD-derived Raman and IR amide I′ profiles reproduce experimental data well, except for the CHARMM Drude-derived anisotropic Raman profile (red curve in Figure S4b).

Similar to the predictions for the central glycine in cationic GGG, CHARMM Drude performs much worse than the Gaussian model, CHARMM36m, and AMOEBA in its ability to capture the spectroscopic data for alanine in cationic GAG in water. While AMOEBA performs better than CHARMM Drude, it does not offer an improvement over CHARMM36m or the Gaussian model in terms of the reproduction of the *J*-coupling constants and amide I′ profiles for alanine in cationic GAG in water.

### Does the C-Terminal Charge Affect Intrinsic Conformational Ensembles of Glycine and Alanine Residues in Water?

The neutral C-terminal cappings of GGG and GAG are used in the simulations described above to facilitate the comparison to the *J*-coupling constants acquired at acidic pH. Because we used different neutral C-terminal groups in CHARMM Drude (COOH) and AMOEBA (CONME),^a^ we performed additional simulations to compare the two polarizable force fields with respect to the conformational manifolds of the central glycine in GGG and alanine in GAG using the same negatively charged C-terminal group, COO^–^. Experimental data on GAG and AAA in water and MD simulations reported in an earlier study suggest that the C-terminal charge associated with COO^–^ versus COOH has no significant effect on the conformational ensemble of alanine.^59^

**Figure 5:**
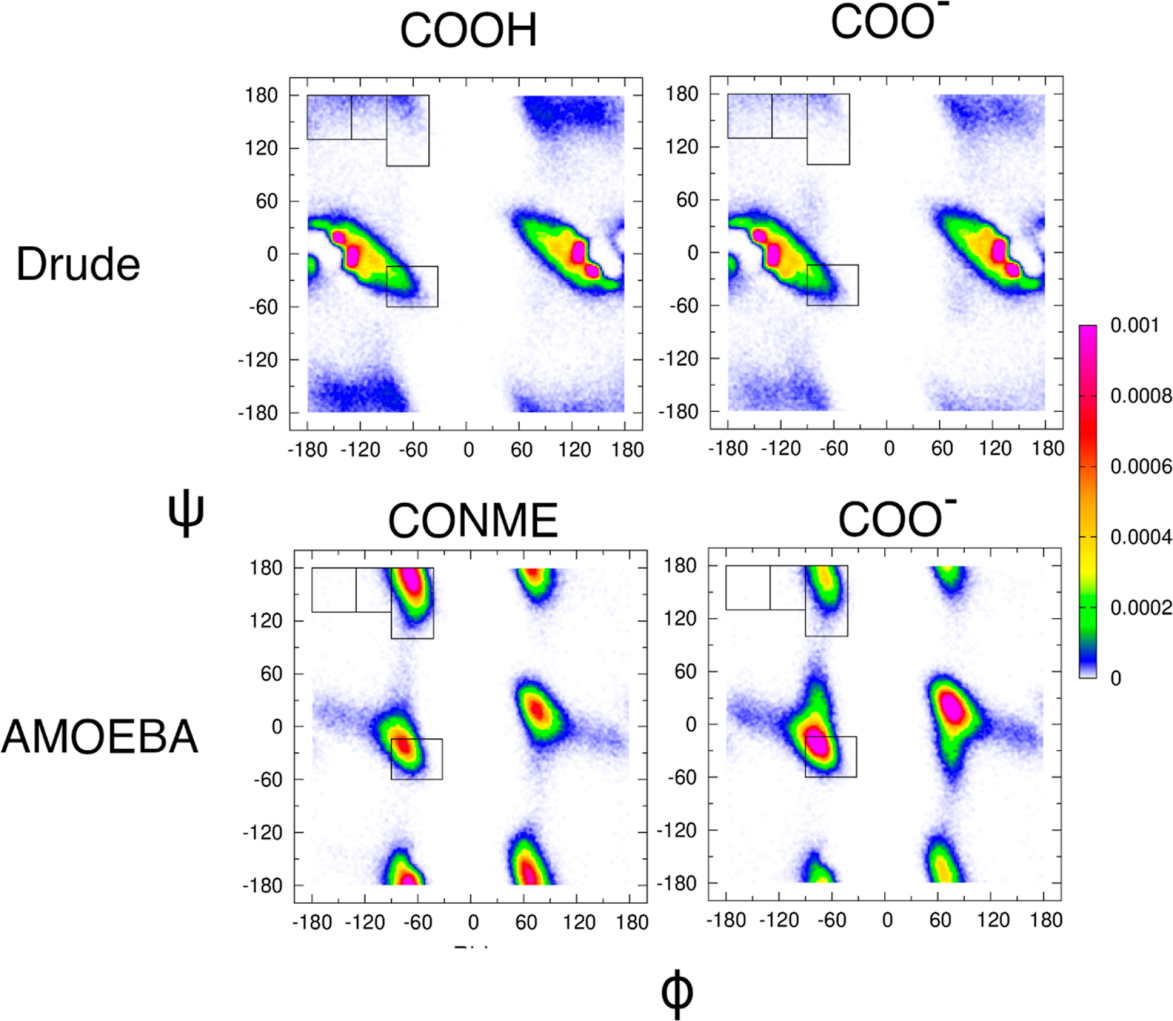
Ramachandran distributions of the central glycine residue in GGG derived within CHARMM Drude (top row) and AMOEBA (bottom row) using neutral (left column) versus negatively charged (right column) C-termini, respectively. The rectangular boxes correspond to the four mesostates as defined in Methods.

Figure 5 shows a comparison of Ramachandran distributions of the central glycine in GGG with a neutral C-terminus (left column) and a negatively charged C-terminus (right column) obtained from CHARMM Drude (top row) and AMOEBA (bottom row) simulations. The corresponding mesostate populations are reported in Table S1. The Ramachandran distributions of the central glycine residue obtained using the two different C-terminal cappings of GGG within CHARMM Drude are very similar, indicating that the negative charge at the C-terminus has no significant effect on the intrinsic conformational ensemble of the glycine residue in GGG in water. Regardless of the C-terminal capping, mesostates near ψ = 0 dominate the Ramachandran distribution, which is at odds with the experimental data. The Ramachandran distributions of the central glycine residue obtained using the two different C-terminal cappings of GGG within AMOEBA are quite different from each other. Replacing the C-terminal CONME capping by COO^–^ results in a sharp increase in the α-helical population from 6 to 17% at the expense of a decrease in the pPII population from 55 to 41%.

**Figure 6:**
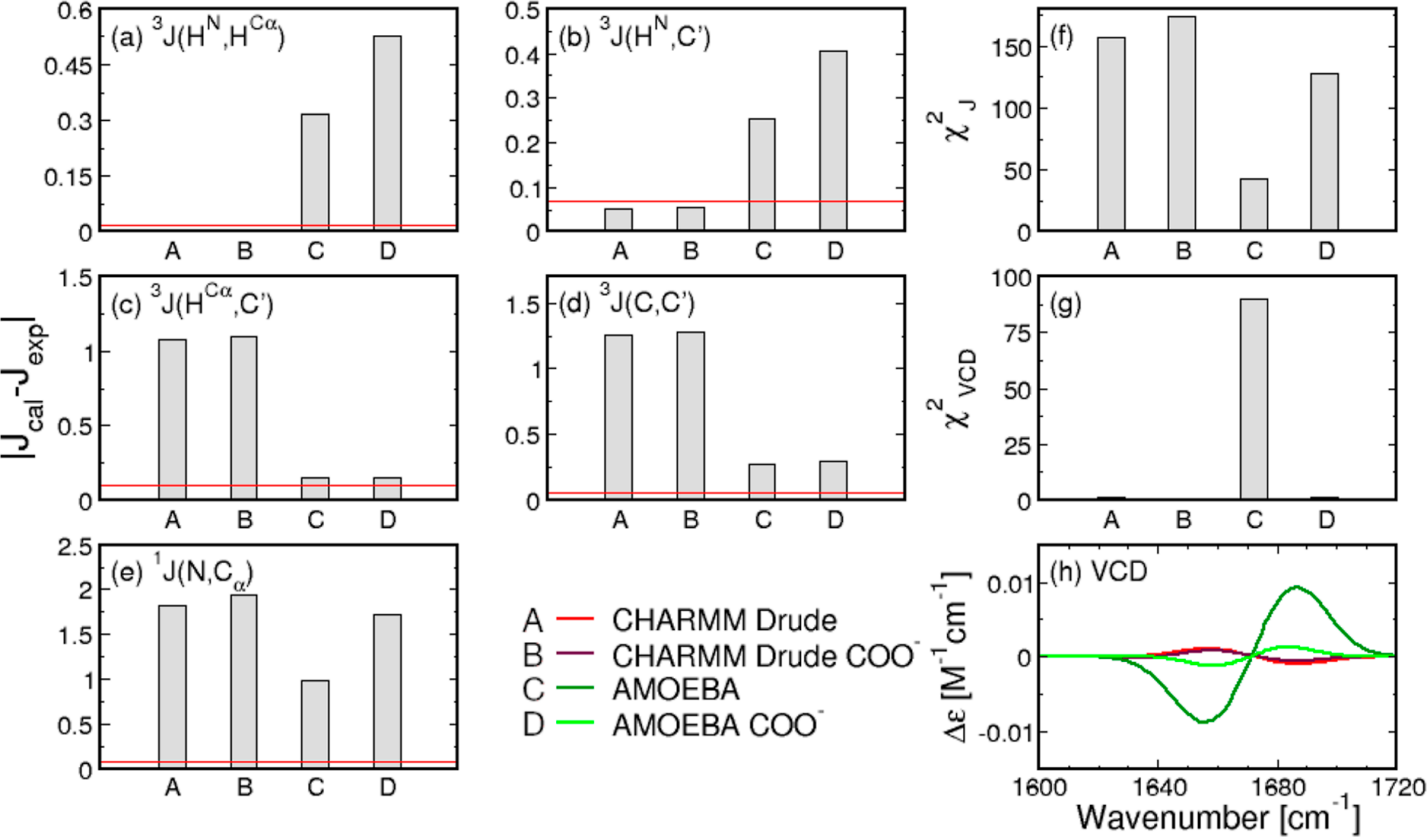
Comparison between experimental and calculated *J*-coupling constants and VCD amide I′ profiles for the central glycine residue in GGG. Comparisons here are made between MD-derived Ramachandran distributions with neutral (COOH or CONME) and charged (COO^–^) C-terminal cappings. (a–e) Absolute differences between calculated and experimental values of the five *J*-coupling constants for the Gaussian model and the two polarizable MD force fields. Red lines correspond to experimental uncertainties. (f,g) χ_*J*_^2^ and χ_VCD_^2^ values for the Gaussian model and MD force fields. (h) VCD amide I′ profiles calculated from the Gaussian and MD-derived Ramachandran distributions.

Figure 6a–f shows a comparison between MD-derived and experimental *J*-coupling constants and reduced χ_*J*_^2^ values for the central glycine residue in GGG for both polarizable force fields and the two C-terminal cappings. The data for each force field with the neutral C-terminus are the same as in Figure 2. For CHARMM Drude, these results show that the difference between the calculated and experimental *J*-coupling constants is not strongly affected when COOH is replaced by COO^–^, which is consistent with the comparable χ_*J*_^2^ values (Figure 6g). For AMOEBA, replacing the neutral CONME capping at the C-terminus by COO^–^ results in significantly larger deviations from the respective experimental values and hence an increased χ_*J*_^2^ value (Figure 6f). MD-derived VCD amide I′ profiles for the central glycine residue in GGG are displayed in Figure 6h. No significant changes in the CHARMM Drude-derived VCD amide I′ profile are observed due to a different C-terminal capping, and, consequently, the χ_VCD_^2^ values are almost the same. Surprisingly, the AMOEBA-derived VCD amide I′ profile of the central glycine residue in GGG with a charged C-terminus exhibits peaks with magnitudes that are much smaller than those observed for the same residue in GGG with the CONME capping (Figure 6h, compare the red and green curves). The experimental and calculated Raman and IR amide I′ profiles for glycine in GGG are shown in Figure S5 for both CHARMM Drude and AMOEBA. Similar to the results for VCD amide I′ profiles, there are no significant differences in CHARMM Drude-derived Raman and IR amide I′ profiles for guest glycine residue in GGG when the neutral C-terminus is replaced by the negatively charged C-terminus, whereas replacing the neutral capping with the negatively charged C-terminus in GGG significantly affects the AMOEBA-derived Raman and IR amide I′ profiles of the guest glycine residue.

**Figure 7:**
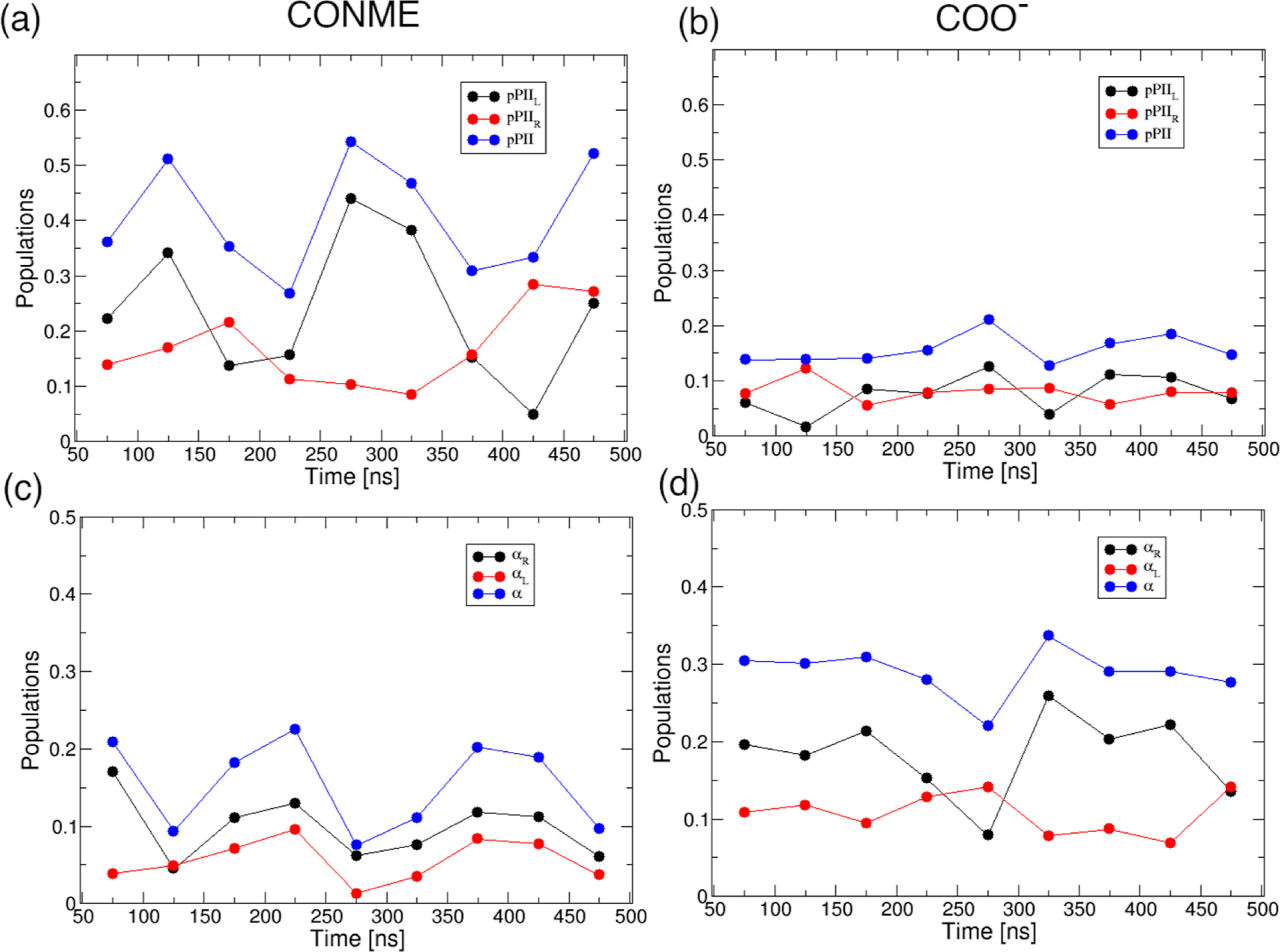
Time evolution of AMOEBA-derived right-handed (ϕ < 0, black), left-handed (ϕ > 0, red), and total (blue) pPII populations for the central glycine residue in GGG with (a) CONME and (b) COO^–^ C-terminal cappings and right-handed (black), left-handed (red), and total (blue) α-helical populations for the central glycine in GGG with (c) CONME and (d) COO^–^ C-terminal cappings. Each point corresponds to a 50 ns time average.

We then asked if the origin of the large magnitude of AMOEBA-derived VCD amide I′ profiles for the central glycine residue in GGG in the case of CONME C-terminal capping may be due to a poor sampling of the Ramachandran space. To this end, we calculated 50 ns long running averages of the right- and left-handed pPII and α-helical mesostate populations using both AMOEBA trajectories for GGG with CONME and COO^–^ capping, as shown in Figure 7. A comparison of the black and red curves in Figure 7, corresponding to right-handed and left-handed mesostate populations, respectively, immediately shows that the right-handed region of the Ramachandran space is sampled more than the left-handed region, which would not be expected for achiral glycine residue. This asymmetry is particularly notable in the case of GGG with CONME capping. Figure 7a shows major differences (≈30% maximum difference) between the left- and right-handed pPII populations in the case of CONME capping. The pPII population of the central glycine residue averaged from 50 to 500 ns of simulation time results in a net difference of ≈7%. This difference contributes to the large VCD amide I′ peak magnitudes observed in Figure 2h. There are also minor differences between the right- and left-handed α-helical populations for central glycine with the COO– C-terminal capping (Figure 7d), albeit of a significantly lower magnitude. When averaged from 50 to 500 ns, the difference between the α-helical populations of the central glycine residue in GGG with the COO^–^ capping is ≈ 7%. Nonetheless, the central glycine in GGG with COO^–^ capping produces the VCD amide I′ profile with much smaller peak magnitudes than in the case of the C-terminal CONME capping. This could be explained by the lower maximum difference between the right- and left-handed mesostate populations in the case of C-terminal COO^–^ capping. Whereas the total mesostate populations fluctuate as a function of simulation time (blue curves in Figure 7), there is no apparent drift of their average values with simulation time, indicating that the observed unequal sampling of the right- and left-handed mesostate populations is not due to poor sampling or a lack of convergence of the MD trajectories.

**Figure 8:**
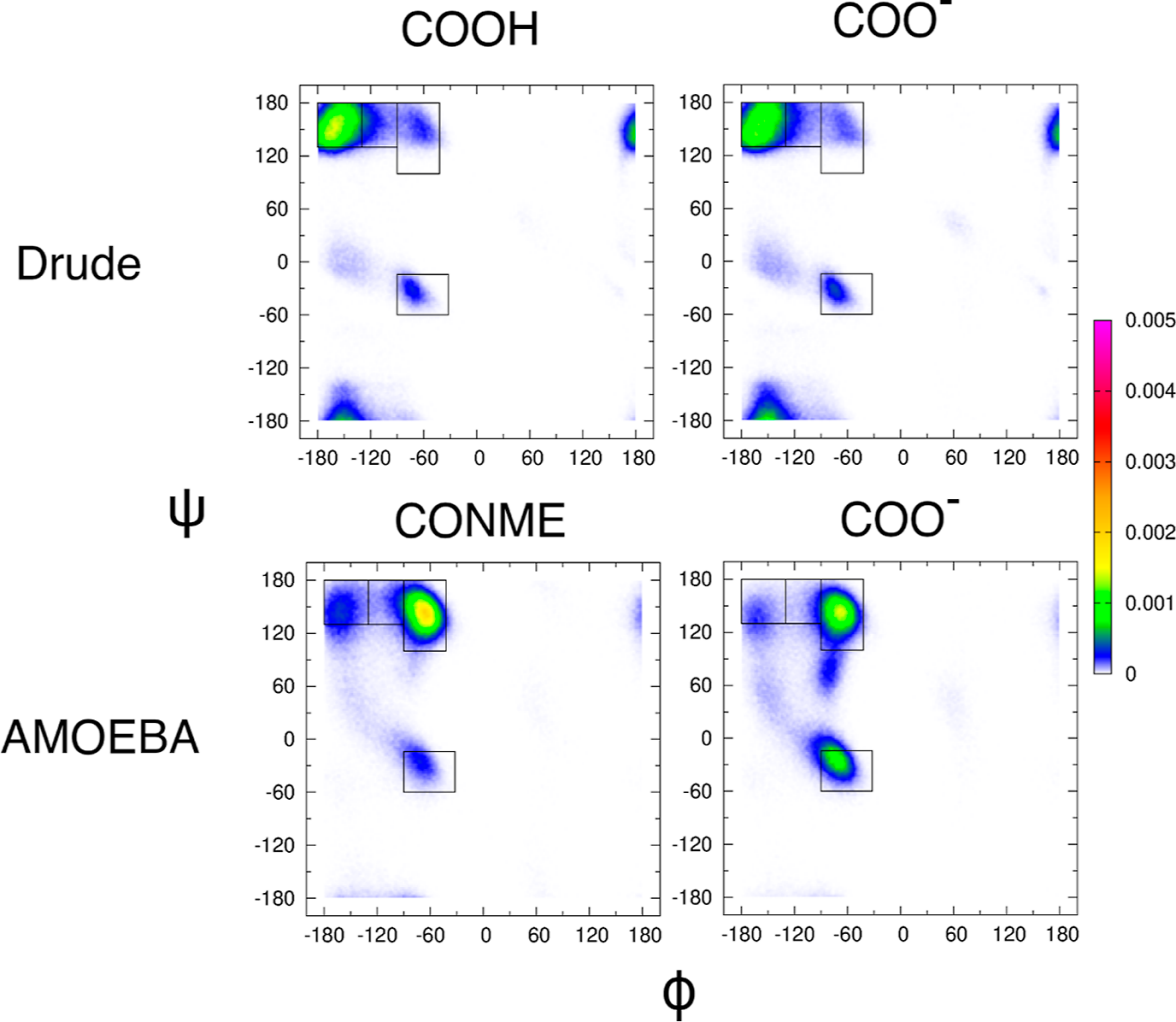
Ramachandran distributions of the alanine residue in GAG obtained within CHARMM Drude (top row) and AMOEBA (bottom row) using neutral (left panels) and charged (right panels) C-terminal cappings. The rectangular boxes correspond to the four mesostates as defined in Methods.

The Ramachandran distributions of the alanine residue in GAG with neutral (left column) and charged (right column) C-terminal cappings derived from the CHARMM Drude (top row) and AMOEBA (bottom row) simulations are displayed in Figure 8, and the respective mesostate populations are tabulated in Table S1. Consistent with the results for the central glycine residue in GGG, the differences in CHARMM Drude-derived Ramachandran distributions are not strongly affected by the C-terminal capping. The population in the aβ region of the Ramachandran distribution slightly decreases when the neutral C-terminus (COOH) is replaced by the charged C-terminus (COO^–^). On the other hand, the AMOEBA-derived Ramachandran distribution of the alanine residue in GAG with a charged C-terminus (COO^–^) exhibits an increased α-helical population at the expense of the pPII and aβ populations relative to the case of the neutral C-terminal capping (CONME). The effect of the C-terminal capping in AMOEBA for the alanine residue in GAG is consistent with the one observed for the central glycine residue in GGG in Figure 5.

**Figure 9:**
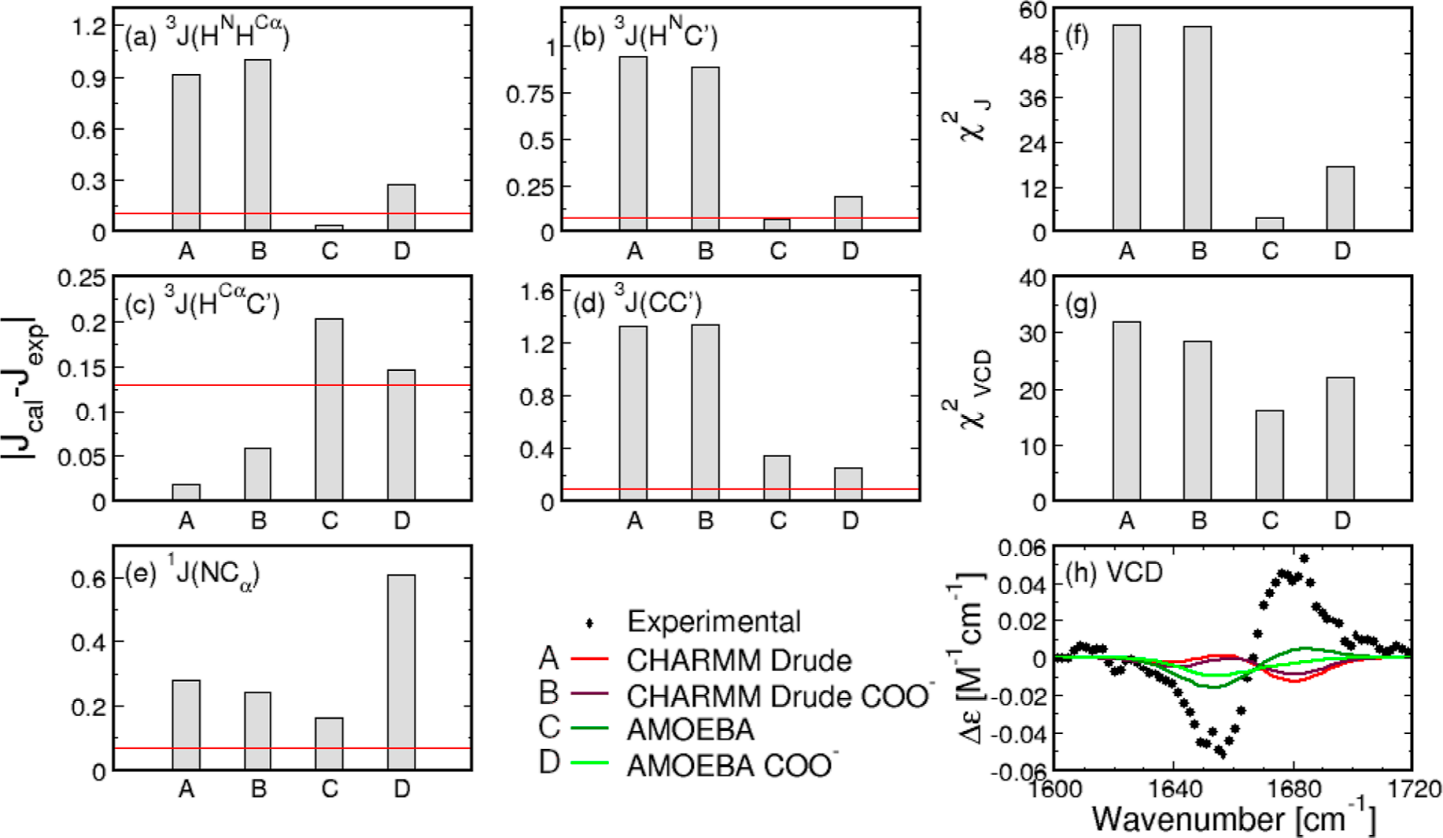
Comparison between experimental and calculated *J*-coupling constants and amide I′ profiles for the alanine residue in GAG. Comparisons here are made between MD-derived Ramachandran distributions for GAG with neutral (COOH or CONME) and charged (COO^–^) C-terminal cappings. (a–e) Absolute differences between the calculated and experimental values of the five *J*-coupling constants for the Gaussian model and the MD force fields. Red lines correspond to experimental uncertainties. (f,g) χ_*J*_^2^ and χ_VCD_^2^ values for the Gaussian model and MD force fields. (h) VCD amide I′ profiles calculated from the Gaussian and MD-derived Ramachandran distributions.

Figure 9a–g shows a comparison between MD-derived and experimental *J*-coupling constants and reduced χ_*J*_^2^ values for the alanine residue in GAG for both force fields and both C-terminal cappings. The data for each force field with the neutral C-terminus are the same as in Figure 4.

As expected based on the comparison of the corresponding Ramachandran distributions, the differences between experimental and MD-derived *J*-coupling constants within CHARMM Drude due to two different C-terminal groups in GAG are minor. Using a charged C-terminus in GAG within CHARMM Drude somewhat improves the correspondence with experimental ^3^*J*(*H*^*N*^, *C*′) and ^1^*J*(*N*, *C*_α_) values. This contrasts with the results for the central glycine residue in GGG, where CHARMM Drude produces larger deviations from the experimental data for all *J*-coupling constants when the neutral C-terminus is replaced by a negatively charged C-terminus. In the case of the alanine residue in GAG, the CHARMM Drude-derived reduced χ_*J*_^2^ value for the alanine residue decreases when the neutral C-terminus is replaced by the charged C-terminus. However, both CHARMM Drude-derived χ_*J*_^2^ values for the two C-terminal cappings of GAG are very large compared to the corresponding value of the Gaussian model. AMOEBA simulations of GAG with the charged C-terminus produce the *J*-coupling constants for the alanine residue that deviate from the experimental values more than the values obtained in the case of the neutral C-terminus, except for^3^*J*(*H*^*C*_α_^, *C*′) and ^3^*J*(*C*, *C*′) values, which are slightly improved. Consequently, the reduced χ_*J*_^2^ value associated with central alanine in the peptide with the charged C-terminus is larger than the χ_*J*_^2^ value for the case of the neutral C-terminus. Figure 9h shows MD-derived VCD amide I′ profiles for the alanine residue in GAG. Only slight deviations are notable in the VCD amide I′ profiles due to two distinct C-termini. In CHARMM Drude, the reduced χ_VCD_^2^ value is smaller for the peptide with the charged C-terminus relative to the neutral C-terminus, whereas the opposite trend is observed for the reduced χ_VCD_^2^ values of the two distinct C-termini in AMOEBA. As shown in Figure S6, the experimental and MD-derived Raman and IR amide I′ profiles for the alanine residue in GAG are not significantly affected by the C-terminal capping in CHARMM Drude, whereas in AMOEBA, the profiles derived from simulations with the neutral C-terminus are slightly better aligned with experimental data than the profiles from simulations with the negatively charged C-terminus.

In AMOEBA, an increase in the α-helical population is observed for both the central glycine residue in GGG and the alanine residue in GAG when the neutral C-terminus (CONME) is replaced with a negatively charged one (COO^–^). In the case of a negatively charged C-terminus, GGG or GAG have oppositely charged termini, which may result in increased intrapeptide hydrogen bonding between the two termini, leading to the formation of 3_10_ helical structures. To investigate this possibility, the average number of intrapeptide and peptide–water hydrogen bonds for GGG and GAG is calculated within CHARMM Drude and AMOEBA for each neutral and negatively charged C-terminus. The results are presented in Table 1, in which the average values of intrapeptide hydrogen bonds are multiplied by 10^3^ for readability. As expected, the average number of intrapeptide hydrogen bonds is very low because formation of such hydrogen bonds is sterically restricted in tripeptides that lack charged side chain groups. In CHARMM Drude, for both GGG and GAG, an approximately 2-fold increase in each of the average number of intrapeptide and water–peptide hydrogen bonds is observed when replacing the neutral C-terminus with a charged C-terminus. This relatively substantial increase in the average number of HBs, however, exerts only a minor effect on the Ramachandran distributions of the glycine residue and alanine residue in GGG and GAG, respectively. Replacing the CONME C-terminal capping with COO^–^ in AMOEBA in GGG and GAG, however, results in a minor decrease of the average number of intrapeptide hydrogen bonds and a minor increase in the average number of water–peptide hydrogen bonds. Thus, the increased population within the α-helical mesostate in AMOEBA upon replacing a neutral C-terminus with a charged C-terminus capping cannot be due to the changes in hydrogen bonding propensity. So, why are the conformational ensembles of the central glycine residue in GGG and the alanine residue in GAG so strongly affected by the C-terminal group in AMOEBA simulations? The neutral CONME C-terminal capping is significantly bulkier than the negatively charged COO^–^ group, so it might be possible that the CONME group exerts sterical effects on GGG or GAG. However, if the increase in the α-helical population in Ramachandran distributions were imparted by sterical effects alone, one would expect this increase to be significant only for the alanine residue in GAG and not for the central glycine residue in GGG as it lacks heavy side chain atoms. The above findings show that in AMOEBA, the differences in mesostate populations of the guest amino acid residues in GGG and GAG due to distinct C-termini cannot be explained by the changes in hydrogen bonding propensities or steric effects imparted by CONME capping.

**Table 1:** Average Number of Intrapeptide and Peptide–Water Hydrogen Bonds for GGG and GAG Peptides with Different C-Termini in Water Obtained from CHARMM Drude and AMOEBA Simulations^a^

force field (CT)	intrapeptide HBs (×10^3^)	peptide–-water HBs
glycine		
Drude (COOH)	0.051 ± 0.013	1.745 ± 0.003
Drude (COO^–^)	0.142 ± 0.036	3.253 ± 0.004
AMOEBA (CONME)	0.624 ± 0.061	2.874 ± 0.006
AMOEBA (COO^–^)	0.091 ± 0.019	2.937 ± 0.007
alanine		
Drude (COOH)	0.040 ± 0.027	1.711 ± 0.003
Drude (COO^–^)	0.090 ± 0.037	3.183 ± 0.006
AMOEBA (CONME)	1.790 ± 0.129	2.751 ± 0.003
AMOEBA (COO^–^)	0.115 ± 0.022	2.803 ± 0.005

a The error bars correspond to the SEM values (see Methods).

The average number of peptide–water hydrogen bonds reported in Table 1 is lower than that previously reported for simulations of GGG and GAG in nonpolarizable force fields.^39^ To elucidate the effect of hydrogen bonding on the guest glycine and alanine residues in GGG and GAG, respectively, the average number of guest residue–water hydrogen bonds is calculated alongside the respective averages associated with each mesostate: pPII, β (combining βt and aβ), and α-helical (Table S3). Both polarizable force fields produce, on average, significantly fewer guest residue–water hydrogen bonds than CHARMM36m and other nonpolarizable force fields studied previously.^38,39^ The average number of guest residue–water hydrogen bonds per mesostate reported in Table S3 and displayed in Figure S7 demonstrate that, with the exception of AMOEBA results for guest glycine in GGG with the CONME capping, the pPII mesostate is associated with the highest average number of guest residue–water hydrogen bonds, consistent with previous findings on additive force fields.^38,39^ These results add to the body of work that elucidates the pPII state as enthalpically stabilized and water-mediated.^60−62^ While it is unclear why the two polarizable force fields produce, on average, fewer peptide–water hydrogen bonds than CHARMM36m and other additive force fields, this result implies that solubility of short unfolded peptides and IDPs is likely underestimated in CHARMM Drude and AMOEBA.

## Conclusions

MD is a powerful tool for exploring the dynamics of proteins and polypeptides at atomistic resolution that complements experimental information. However, the utility of MD depends on the accuracy of the underlying MD force field. There are multiple issues that existing MD force fields have not resolved yet.^18,63,64^ We here focus on the recently reported inability of additive (nonpolarizable) MD force fields to capture the intrinsic conformational preferences of guest amino acid residues x in cationic GxG peptides in water in agreement with spectroscopic data, which indicate a high degree of amino acid residue-specificity of intrinsic Ramachandran distributions and, in particular, residue-specific pPII-β balance.^37,60^ We expect that the errors in capturing the intrinsic conformational ensembles of amino acid residues in water will propagate to longer unfolded peptides and affect the reliability of conformational dynamics predictions for IDPs.

We here ask if the inclusion of polarization effects improves MD predictions with respect to the intrinsic conformational preferences of amino acid residues in water. To this end, we examine the ability of two distinct polarizable MD force fields, CHARMM Drude 2019 and AMOEBA 2018, to reproduce the experimental constraints associated with the intrinsic conformational dynamics of guest glycine and alanine in cationic GxG peptides in water. Each of these two polarizable force fields implements polarization effects in a distinct and unique way, either in the form of Drude masses on a spring (CHARMM Drude) or through explicit calculations of multipole forces (AMOEBA). In both cases, the inclusion of polarizability effects is expected to reduce the computational efficiency of MD simulations at the expense of improved accuracy. We acquired 500 ns-long MD trajectories of various GGG and GAG systems in CHARMM Drude and AMOEBA. The resulting Ramachandran distributions were then quantitatively compared to a comprehensive set of experimental data including 5 *J*-coupling constants and VCD amide I′ profiles using reduced χ_*J*_^2^ and χ_VCD_^2^ values. Experiment-based Gaussian model Ramachandran distributions for the central glycine residue in GGG and the alanine residue in GAG, which were optimized to fit the above spectroscopic data, as reported previously,^33^ were used as a benchmark for the comparison to MD-derived Ramachandran distributions. The Ramachandran distributions obtained within CHARMM Drude and AMOEBA are compared to those derived within the additive MD force field, CHARMM36m,^19^ which performs relatively well in comparison to other additive MD force fields examined in recent studies.^23,37−39^ The results of this study show that for both guest glycine and alanine residues in GxG peptides in water, CHARMM Drude performs much worse than AMOEBA, and neither of the two polarizable force fields shows any improvement over the additive force field CHARMM36m. Notably, the average number of hydrogen bonds between the guest residue and water is significantly lower in AMOEBA and even more so in CHARMM Drude than in CHARMM36m and other additive force fields that were examined previously,^38,39^ which may directly affect the solubility of unfolded peptides and IDPs.

For the guest glycine residue, the conformational ensembles of the CHARMM Drude-derived Ramachandran distribution are restricted almost exclusively to the region around ψ = 0 with almost no conformations within the pPII region, which contradicts the experiment-based Gaussian model^39^ and is reflected in a large χ_*J*_^2^ value. For the guest alanine residue, the CHARMM Drude-derived Ramachandran distribution is dominated by the aβ mesostate, contrary to many experimental, MD, and DFT studies.^28,38,61,65−67^ This is somewhat surprising because the backbone dihedral angle distributions in CHARMM Drude are optimized on target data for polyalanine ALA_5_, for which the experimental studies reported a predominant sampling of the pPII basin alongside a significant helical population.^27,68^ Although CHARMM Drude clearly does not account for the dominant conformational population of these two residues, i.e., the pPII state, it is noteworthy that upon replacing the neutral C-terminus (COOH) by a negatively charged one (COO^–^), the Ramachandran distributions of the guest glycine and alanine residues in GGG and GAG, respectively, do not change significantly despite the approximately 2-fold increase in the affinity for intrapeptide and water–peptide hydrogen bonding. This insensitivity of the intrinsic conformational ensembles of guest glycine and alanine residues in GxG peptides in water is in line with experimental observations reported by Toal and collaborators,^59^ as discussed in more detail below.

While capturing the intrinsic conformational dynamics of glycine significantly better than CHARMM Drude, AMOEBA performs worse than the additive MD force field CHARMM36m and the Gaussian model. AMOEBA simulations for the guest glycine residue in GGG result in a higher χ_*J*_^2^ value than that of CHARMM36m simulations and the Gaussian model and also produce the highest χ_VCD_^2^ value among the three force fields. The high χ_*J*_^2^ value for AMOEBA stems from large errors in ^3^*J*(*H*^*N*^, *C*′) and ^1^*J*(*N*, *C*_α_) coupling constants, which are likely due to a large preference of the guest glycine residue for α-helical conformations. AMOEBA also produces a high χ_VCD_^2^ value stemming from the high amplitude peaks in the VCD amide I′ profiles. Further examination revealed that AMOEBA produces unequal populations of left- and right-handed pPII conformations for the guest glycine in cationic GGG. This unequal sampling leads to peak magnitudes in the VCD amide I′ profile that are large enough to be detected experimentally. This is problematic because the VCD amide I′ profile is expected to be zero for any achiral system. To the best of our knowledge, no experimentally detectable amide I′ profile has been reported in the literature for the guest glycine residue in GGG. These large peak magnitudes in the VCD amide I′ profile diminish when the neutral C-terminus (CONME) is replaced by a negatively charged C-terminus (COO^–^). This change of the C-terminus also increases the α-helical population at the expense of the pPII population, in disagreement with the experimental findings of Toal et al., which showed that the conformational dynamics of short peptides is mostly unaffected by the charged state of the C-terminus.^59^ An earlier study by Avbelj and collaborators also reported that the experimentally determined ^3^*J*(*H*^*N*^, *H*^*C*_α_^) coupling constant was the same for blocked dipeptides and guest residues x in GGxGG peptides.^69^ A more recent comparison by Schweitzer-Stenner et al. showed that the same J-coupling constant in blocked dipeptides and corresponding guest residues x in GxG peptides are consistent with each other.^25^ These results together support the expectation that the C-terminal charge should not significantly affect the intrinsic conformational preferences of the guest amino acid residues in water.

In the case of the alanine residue in GAG, AMOEBA performed slightly worse than CHARMM36m by overestimating the α-helical population, which produces a large deviation from the experimental ^1^*J*(*N*, *C*_α_) value, similar to the case of the guest glycine residue in GGG. As expected, the Ramachandran distribution of the alanine residue in GAG in this study is consistent with the respective AMOEBA-derived distribution for alanine dipeptide published by Peng and collaborators.^70^ In our simulations, changing the neutral to a negatively charged C-terminus produces an increase in the α-helical population, which is not in line with experimental findings.^25,59,69^ One could argue that the bulkiness of the neutral CONME capping in our AMOEBA simulations may be the cause of an overestimated α-helical population that makes the comparison to experimental data less favorable; however, this does not seem to be the case because this population becomes even more overestimated in the presence of a negatively charged C-terminus, COO^–^. Interestingly, in AMOEBA, the water–peptide hydrogen bonding is a lot less affected by the C-terminal CONME to COO^–^ substitution than in the case of the C-terminal COOH to COO^–^ substitution in CHARMM Drude, yet the corresponding changes imparted on the conformational ensembles of the guest glycine and alanine residues in AMOEBA are significant.

It should be noted that the rather low population of right-handed helical conformations predicted by the Gaussian analysis is consistent with the σ-values in the range between 2.5 × 10^–3^ and 3.5 × 10^–3^ obtained from the Zimm–Bragg theory applied to thermal unfolding and folding of alanine-based peptides.^71^ In the Zimm–Bragg theory, this parameter reflects the statistical weight of a helical residue that is not incorporated in a segment stabilized by the backbone hydrogen bonding. The respective numbers should therefore be comparable to the mole fraction of the right-handed helical population within the Gaussian analysis. The helical propensities that emerge from AMOEBA simulations thus overestimate the σ-value by nearly an order of magnitude.

In summary, our study shows that the two polarizable force fields, CHARMM Drude and AMOEBA, do not offer an improved description of the intrinsic conformational ensembles of guest glycine and alanine residues in GxG peptides in water over the additive MD force field CHARMM36m. Considering that these two polarizable force fields were validated mostly on folded proteins rather than IDPs, these results may not be too surprising. However, the application of CHARMM Drude and AMOEBA to IDPs may be limited without resolving the issues reported here. Results of this work and several previous studies^37−39^ demonstrate that the experimental data set for MD force field calibration needs to be complete enough to sufficiently constrain the Ramachandran distribution with respect to both dihedral angles, ϕ and ψ, to avoid introducing a bias into the force field. Our findings also elucidate the importance of triglycine as a model of a peptide backbone; a failure to capture the correct conformational dynamics of the guest glycine residue in GGG in water will affect the conformational ensembles of all other amino acid residues in water. The question that needs to be addressed in future studies is whether a proper optimization of the dihedral potentials in CHARMM Drude and AMOEBA alone would be sufficient to produce the intrinsic Ramachandran distributions of glycine and alanine residues in water that are consistent with the available experimental data.

## Supporting Information Available

Input files and scripts for MD simulations in CHARMM Drude and AMOEBA (ZIP)
Mesostate populations of guest amino acid residues considered in this work; experimental and calculated *J*-coupling constants; average hydrogen bonds between the central residues of GGG and GAG and water for the whole simulation and in each mesostate; dihedral angles vs simulation time for simulations of central glycine in GGG; experimental and calculated amide I′ profiles from Raman and IR spectroscopies for the central glycine residue in GGG; dihedral angles vs simulation time for simulations of central alanine in GAG; experimental and calculated amide I′ profiles from Raman and IR spectroscopies for the alanine residue in GAG; experimental and calculated amide I′ profiles from Raman and IR spectroscopies for the central glycine residue in GGG to compare the effect of different C-termini; experimental and calculated amide I′ profiles from Raman and IR spectroscopies for the alanine residue in GAG to compare the effect of different C-termini; and average number of hydrogen bonds between the central residue of GGG and GAG broken down by the mesostate (PDF)

## Footnotes

a The COOH C-terminal capping is not available for AMOEBA in OpenMM.
