# Supplementary material for: Intrinsic Conformational Dynamics of Glycine and Alanine in Polarizable Molecular Dynamics Force Fields: Comparison to Spectroscopic Data
Source: J Phys Chem B. 2024 Jun 15;128(25):6217–31. doi: 10.1021/acs.jpcb.4c02278 (PMC11215781; doi:10.1021/acs.jpcb.4c02278)
Supplement: Supplementary file 2 — jp4c02278_si_003.pdf [file jp4c02278_si_003.pdf]

Supporting Information: Intrinsic Conformational Dynamics of Glycine and Alanine in Polarizable MD Force Fields: Comparison to Spectroscopic Data

Brian Andrews,† Reinhard Schweitzer-Stenner,‡ and Brigita Urbanc*,†

†*Department of Physics, Drexel University, Philadelphia, PA 19104, USA*

‡*Department of Chemistry, Drexel University, Philadelphia, PA 19104, USA*

E-mail: bu25@drexel.edu

Fax: (215) 895-5934

Supporting Tables

Table S1: Mesostate populations of all guest amino acid residues in GxG considered in this work. Populations are calculated using simulation times 50-500 ns. Populations from previous works are calculated using simulation times 50-300 ns.

**Populations**	**pPII**	**βt**	**aβ**	**α**	**pPII**	**βt**	**aβ**	**α**
	**GGG**[a]				**GGG (COO^-)**[a]			
**Gaussian model**[b]	0.46	0.13	0.01	0.06	-	-	-	-
**CHARMM36m**[b]	0.48	0.02	0.01	0.04	0.51	0.02	0.01	0.05
**CHARMM Drude**	0.01	0.01	0.01	0.08	0.01	0.01	0.01	0.05
**AMOEBA**	0.41	0.01	0	0.15	0.15	0.01	0	0.29
	**GAG**				**GAG (COO^-)**			
**Gaussian model**[c]	0.59	0.16	0.02	0.02	-	-	-	-
**CHARMM36m**[c]	0.55	0.09	0.12	0.06	-	-	-	-
**CHARMM Drude**	0.07	0.10	0.52	0.05	0.06	0.09	0.48	0.05
**AMOEBA**	0.55	0.08	0.12	0.06	0.41	0.05	0.05	0.17

[a] Includes both left and right-handed mesostate populations.
[b] Data taken from Andrews *et al.*[S1]
[c] Data taken from Zhang *et al.*[S2]

Table S2: Experimental and calculated J coupling constants and the uncertainty values used in χ_J^2 calculations for all guest amino acid residues in GxG considered in this work. The MD-derived values for each of the three force fields are based on conformations within 50–500 ns of each trajectory. Data from previous papers are calculated using 50-300 ns of simulation time.

	$^3J(H^N,H^{C_\alpha})$	$^3J(H^N,C')$	$^3J(H^{C_\alpha},C')$	$^3J(C,C')$	$^1J(N,C_\alpha)$
		**GGG**			
Experimental	5.89	1.10	4.01	0.26	12.17
Gaussian[a]	5.94	1.16	3.95	0.66	11.78
CHARMM36m[a]	5.99	1.17	3.93	0.60	11.69
CHARMM Drude	5.88	1.05	2.93	1.52	10.34
AMOEBA	6.02	0.85	3.86	0.54	11.18
Uncertainty	0.02	0.07	0.1	0.03	0.07
		**GGG (COO^-)**			
CHARMM36m[a]	6.00	1.63	3.92	0.61	11.69
CHARMM Drude	5.89	1.04	2.91	1.54	10.23
AMOEBA	6.42	0.69	4.16	0.56	10.46
	$^3J(H^N,H^{C_\alpha})$	$^3J(H^N,C')$	$^3J(H^{C_\alpha},C')$	$^3J(H^N,C_\beta)$	$^1J(N,C_\alpha)$
		**GAG**			
Experimental	6.11	1.18	1.90	2.09	11.28
Gaussian[b]	6.00	1.09	1.89	1.95	11.39
CHARMM36m[b]	6.37	1.24	2.05	1.67	11.21
CHARMM Drude	7.01	2.13	1.92	0.76	11.56
AMOEBA	6.14	1.26	1.69	1.75	11.12
Uncertainty	0.02	0.07	0.1	0.03	0.07
		**GAG (COO^-)**			
CHARMM Drude	7.10	2.08	1.96	0.76	11.52
AMOEBA	6.37	1.00	1.75	1.83	10.67

[a] Data taken from Andrews *et al.*[S1]
[b] Data taken from Zhang *et al.*[S2]

Table S3: Average number of hydrogen bonds between water and guest glycine and alanine residues in GGG and GAG peptides with different C-termini in water obtained from CHARMM Drude and AMOEBA simulations. The average number of guest residue-water HBs associated with pPII, β (βt and aβ combined), and α-helical mesostates are also included. The errors bars correspond to the SEM values.

Force Field (CT)	Guest Residue-Water HBs	pPII	β	α
**Glycine**				
CHARMM36m (COOH)[a]	-	1.28 ± 0.004	1.17 ± 0.002	1.22 ± 0.002
Drude (COOH)	0.807 ± 0.002	0.758 ± 0.013	0.669 ± 0.009	0.743 ± 0.007
Drude (COO^-)	0.916 ± 0.002	0.876 ± 0.029	0.792 ± 0.018	0.814 ± 0.008
AMOEBA (CONME)	0.901 ± 0.003	0.913 ± 0.003	0.901 ± 0.018	0.899 ± 0.004
AMOEBA (COO^-)	1.117 ± 0.003	1.121 ± 0.005	1.100 ± 0.004	1.091 ± 0.003
**Alanine**				
CHARMM36m (COOH)[b]	-	1.3	1.13	1.2
Drude (COOH)	0.746 ± 0.002	0.823 ± 0.006	0.720 ± 0.002	0.796 ± 0.007
Drude (COO^-)	0.833 ± 0.002	0.917 ± 0.006	0.799 ± 0.002	0.875 ± 0.006
AMOEBA (CONME)	0.835 ± 0.002	0.882 ± 0.002	0.664 ± 0.003	0.879 ± 0.006
AMOEBA (COO^-)	1.046 ± 0.002	1.071 ± 0.003	0.894 ± 0.005	1.052 ± 0.004

[a] Data taken from Andrews *et al.*[S1]
[b] Data taken from Zhang *et al.*[S2] No error bars were reported.

Supporting Figures

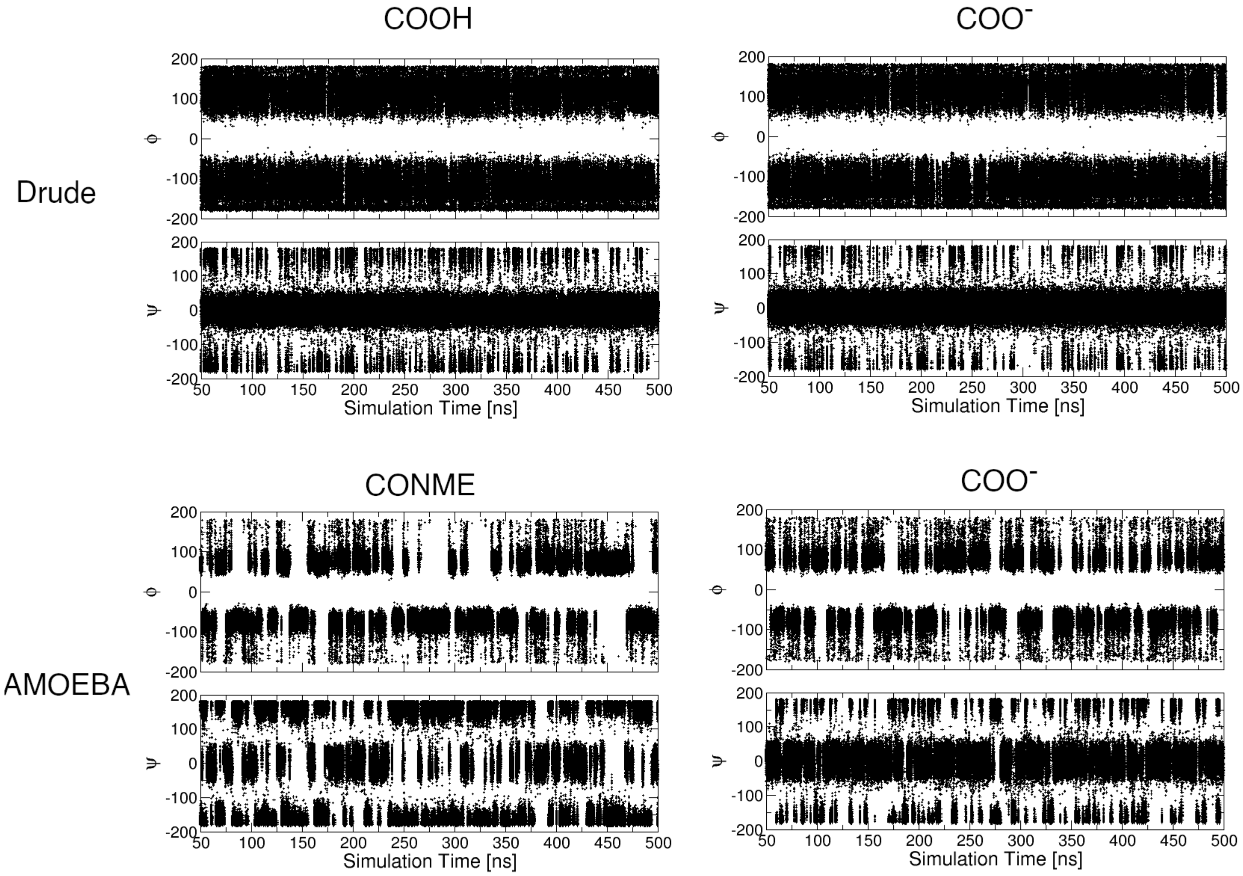

Figure S1: Dihedral angles ϕ and ψ as a function of simulation time for guest glycine residue in CHARMM Drude and AMOEBA simulations of GGG with two different C-termini. Dihedral angles are recorded every 2 ps.

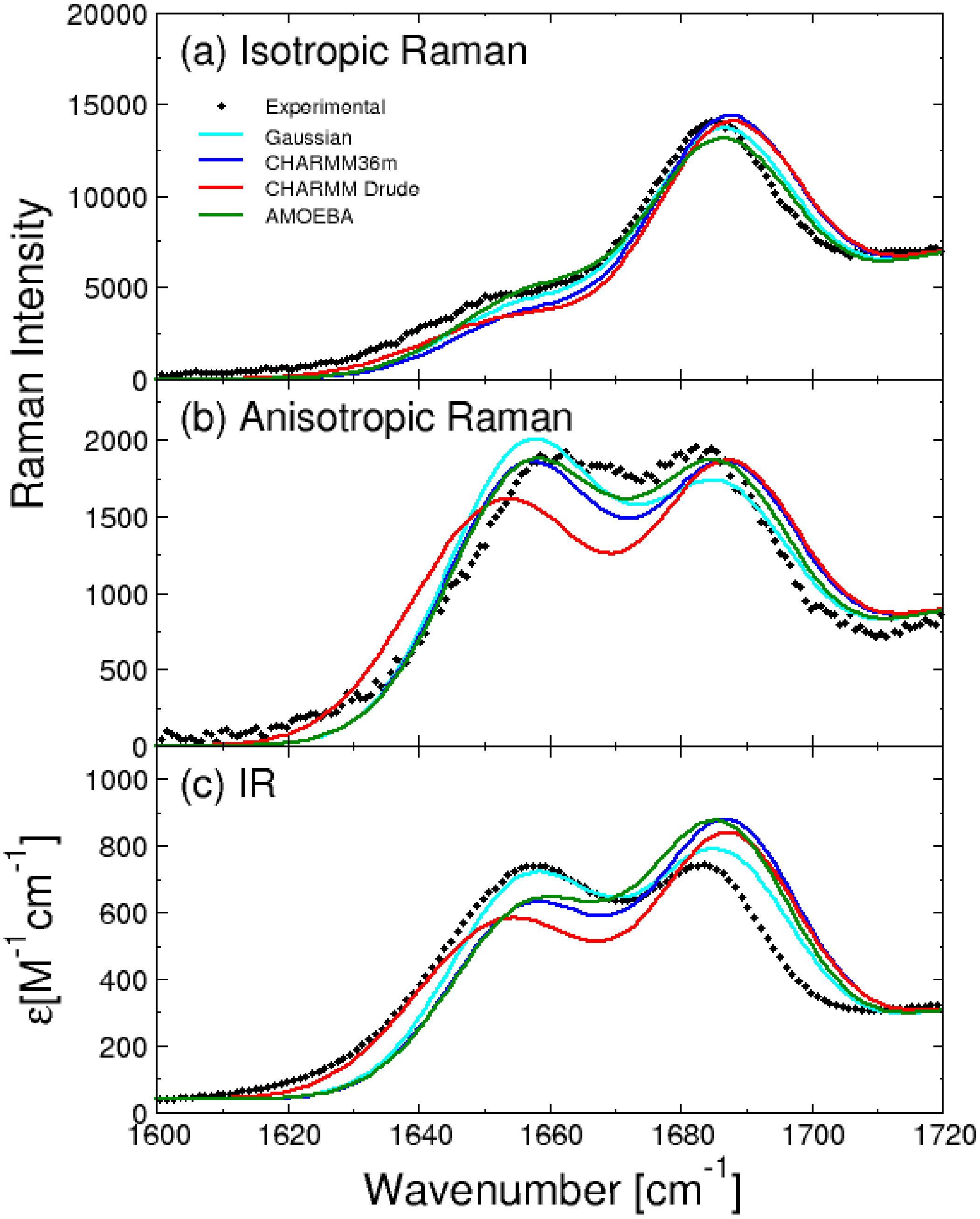

Figure S2: Amide I' profiles for the central glycine residue in GGG. Experimental amide I' profiles derived from (a) isotropic Raman, (b) anisotropic Raman, and (c) IR spectroscopy measurements are compared to predictions of the Gaussian model and MD simulations with CHARMM36m, CHARMM Drude, and AMOEBA.

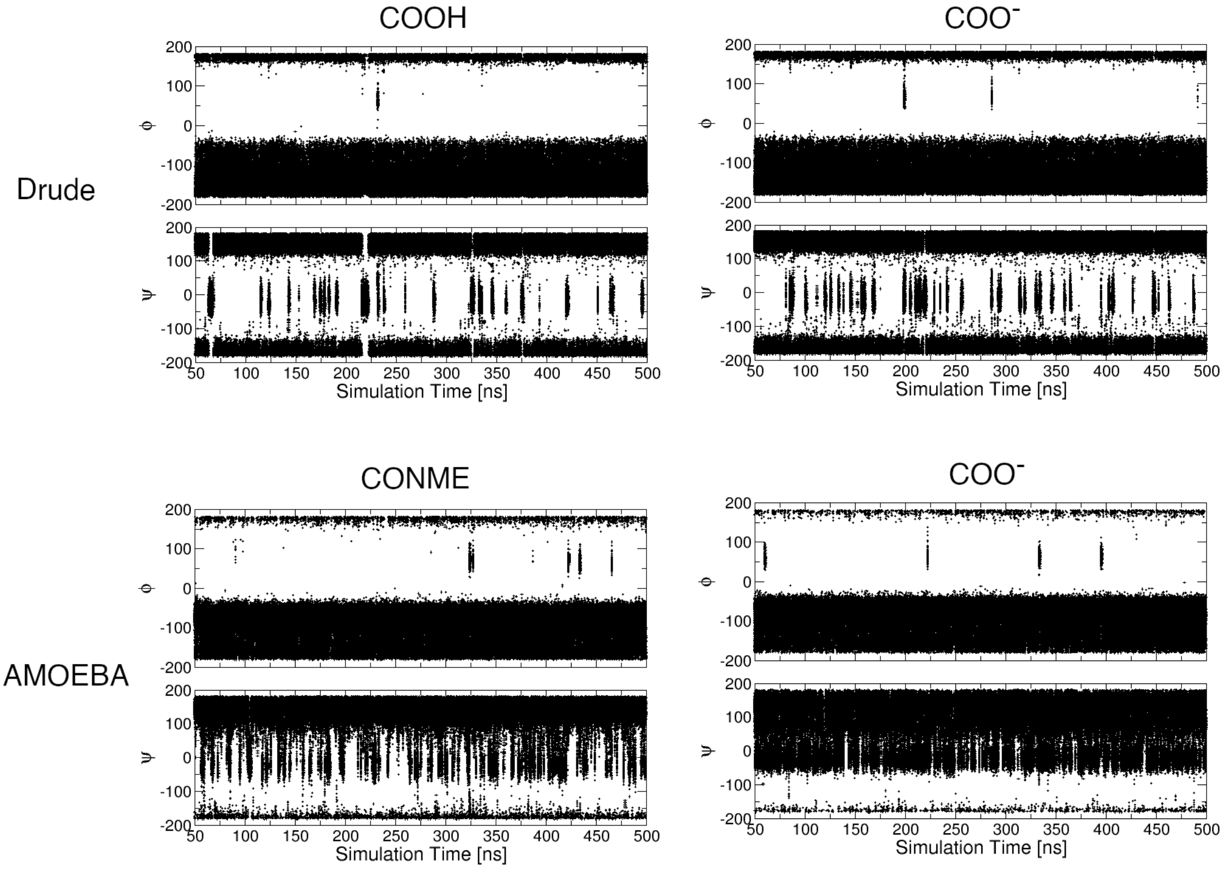

Figure S3: Dihedral angles ϕ and ψ as a function of simulation time for guest alanine residue in CHARMM Drude and AMOEBA simulations of GAG with two different C-termini. Dihedral angles are recorded every 2 ps.

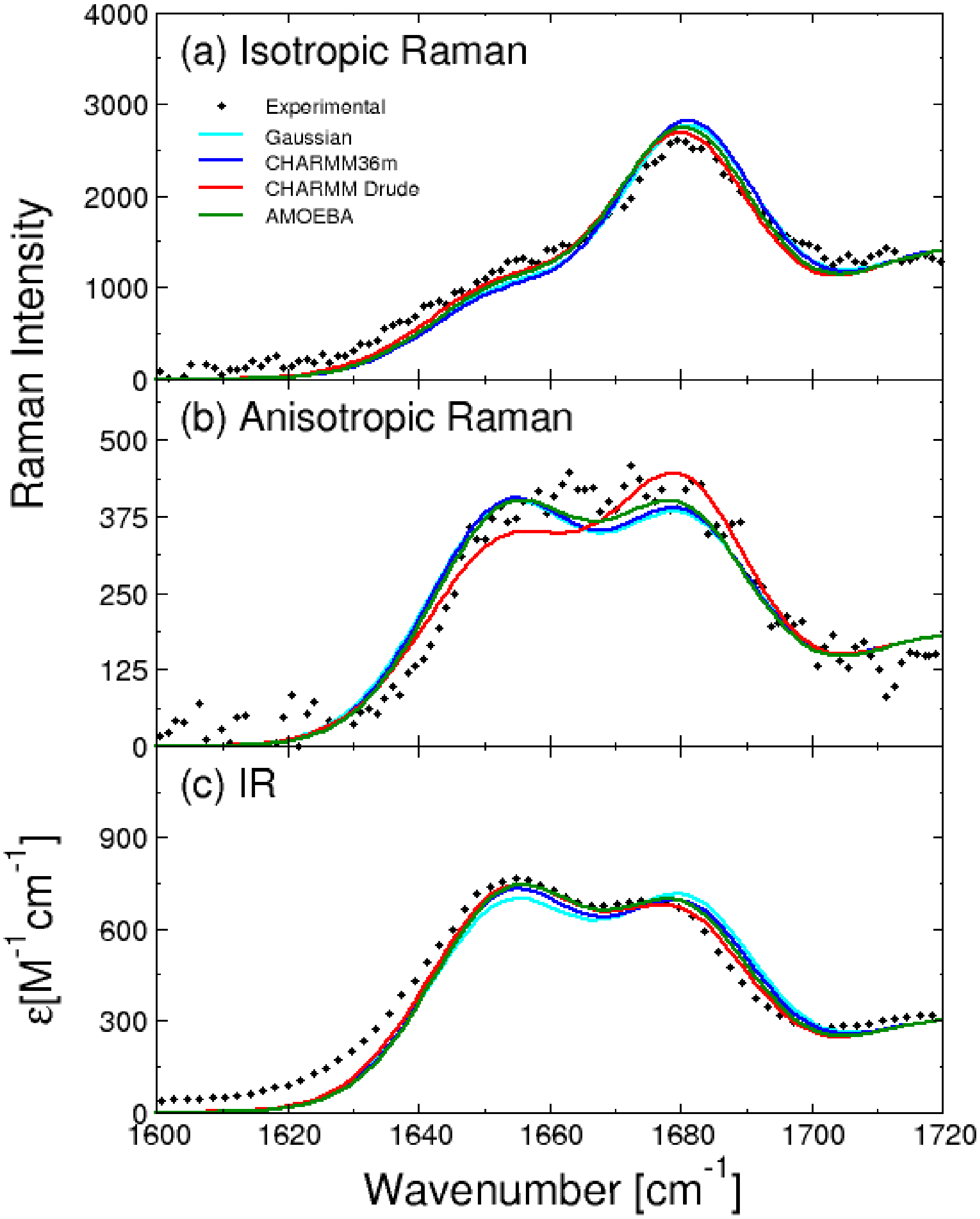

Figure S4: Amide I' profiles for the alanine residue in GAG. Experimental amide I' profiles derived from (a) isotropic Raman, (b) anisotropic Raman, and (c) IR spectroscopy measurements are compared to predictions of the Gaussian model and MD simulations with CHARMM36m, CHARMM Drude, and AMOEBA.

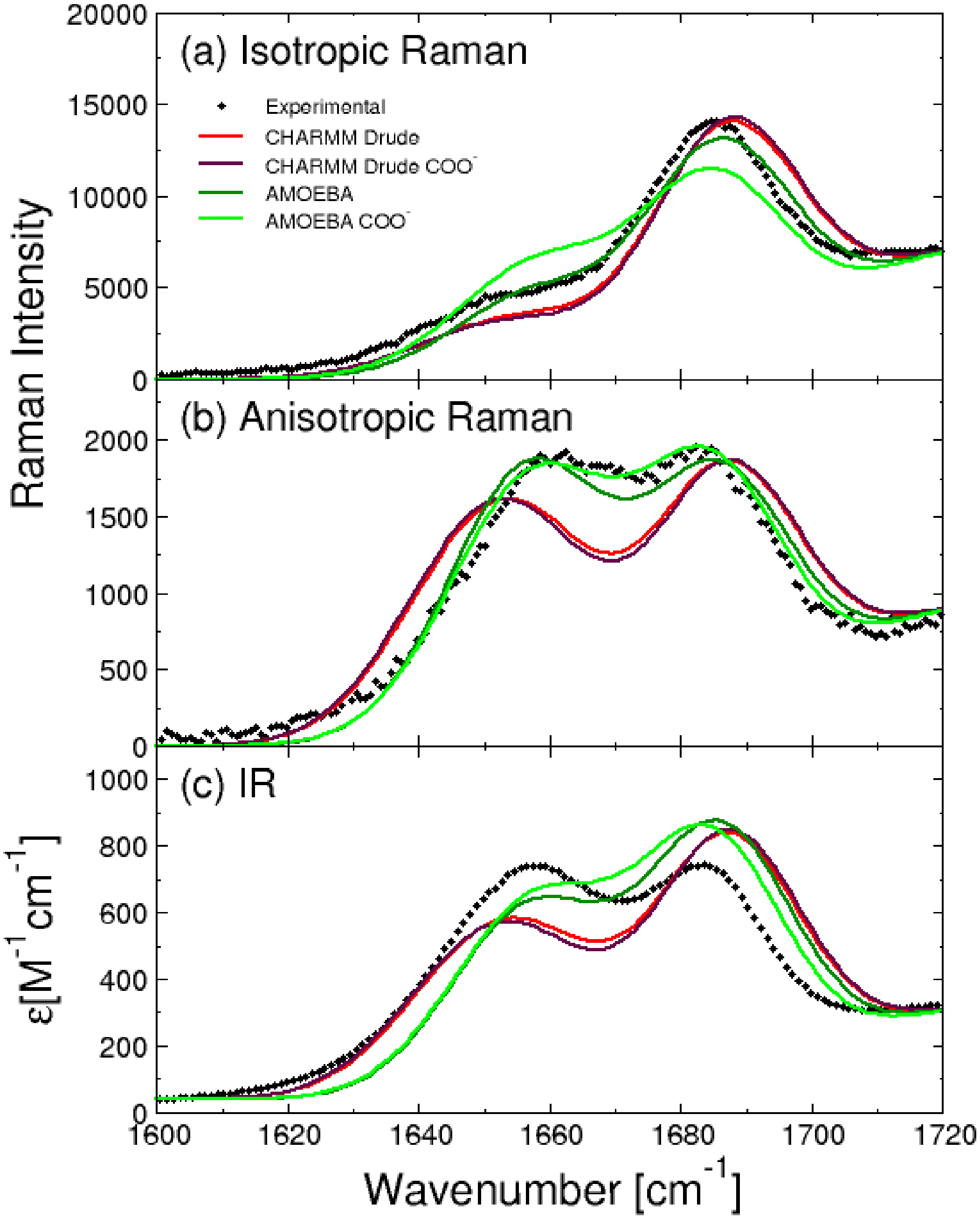

Figure S5: Amide I' profiles for the central glycine residue in GGG. Experimental amide I' profiles derived from (a) isotropic Raman, (b) anisotropic Raman, and (c) IR spectroscopy measurements are compared to predictions MD simulations with CHARMM Drude and AMOEBA with neutral (COOH or CONME) and charged (COO^-) C-termini.

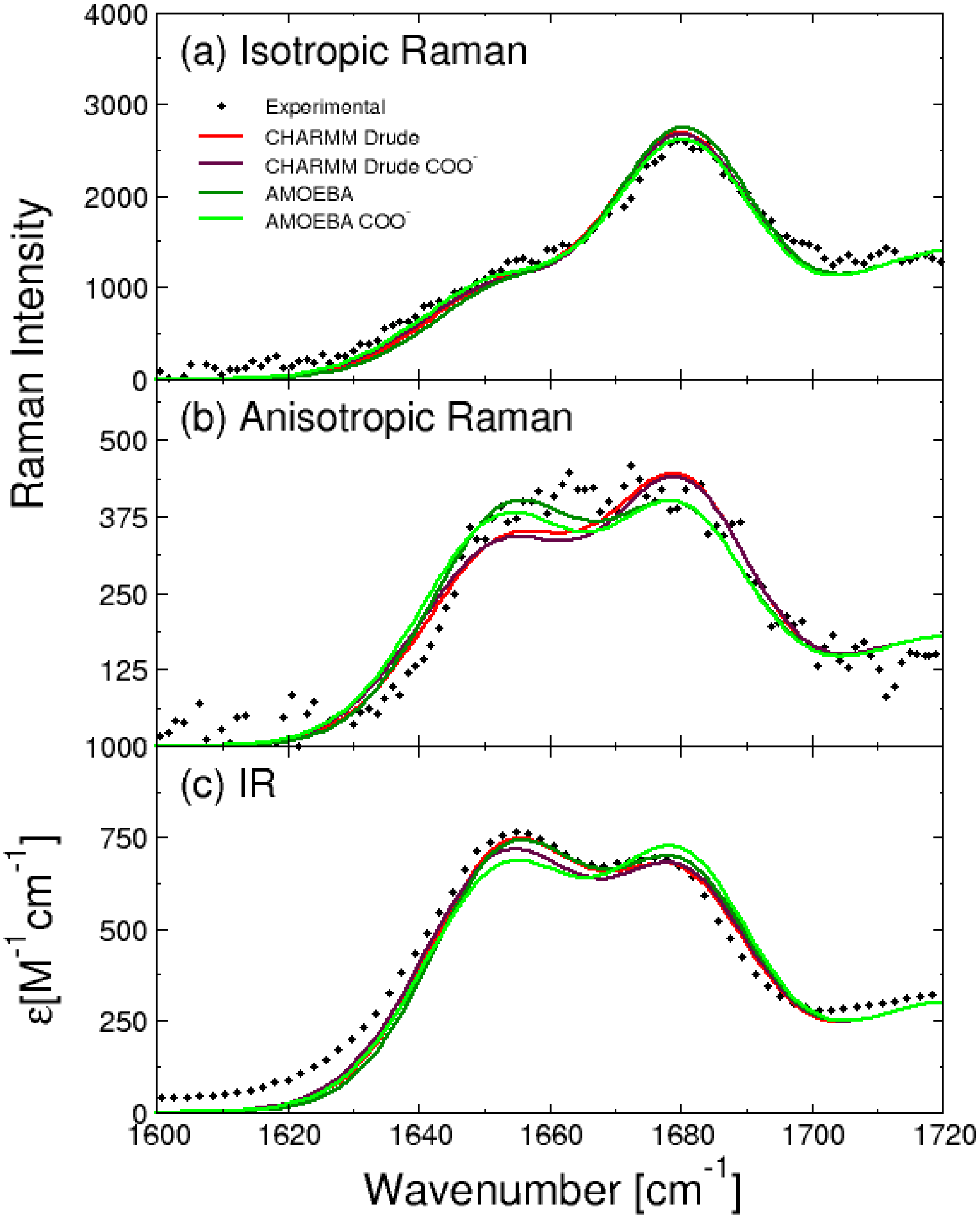

Figure S6: Amide I' profiles for the alanine residue in GAG. Experimental amide I' profiles derived from (a) isotropic Raman, (b) anisotropic Raman, and (c) IR spectroscopy measurements are compared to predictions MD simulations with CHARMM Drude and AMOEBA with neutral (COOH or CONME) and charged (COO^{-}) C-termini.

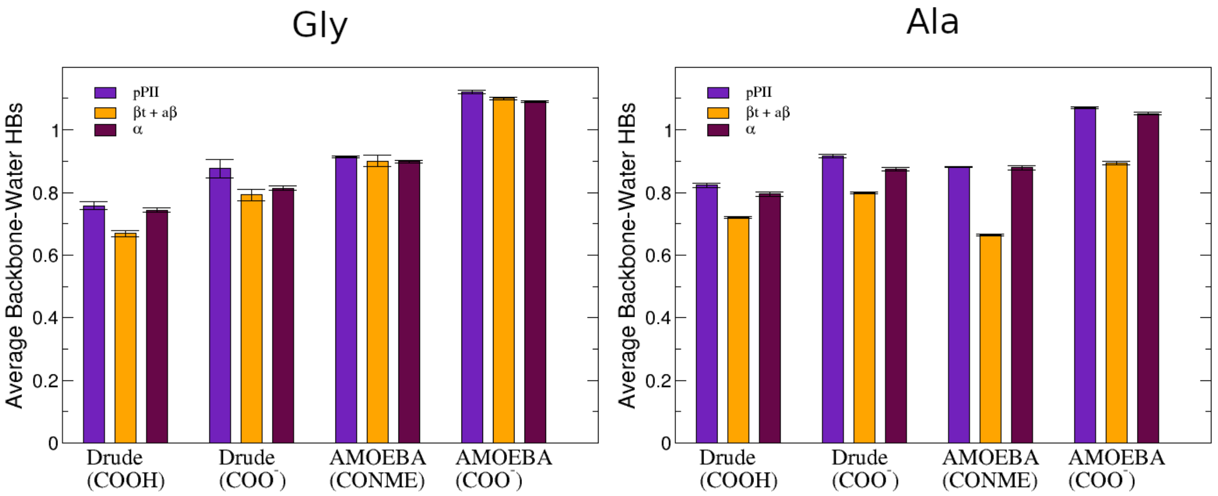

Figure S7: Average number of hydrogen bonds between water and guest glycine residue in GGG (left graph) or guest alanine residue in GAG (right graph). Error bars correspond to the SEM values.

References

(S1) Andrews, B.; Zhang, S.; Schweitzer-Stenner, R.; Urbanc, B. Glycine in water favors the polyproline II state,. *Biomolecules* **2020**, *10*, 1121.

(S2) Zhang, S.; Schweitzer-Stenner, R.; Urbanc, B. Do molecular dynamics force fields capture conformational dynamics of alanine in water? *J. Chem. Theory Comput.* **2020**, *16*, 510–527.
